# Interplay between the cyclophilin homology domain of RANBP2 and MX2 regulates HIV-1 capsid dependencies on nucleoporins

**DOI:** 10.1128/mbio.02646-24

**Published:** 2025-01-24

**Authors:** Haley Flick, Ananya Venbakkam, Parmit K. Singh, Bailey Layish, Szu-Wei Huang, Rajalingam Radhakrishnan, Mamuka Kvaratskhelia, Alan N. Engelman, Melissa Kane

**Affiliations:** 1 Department of Pediatrics, Division of Infectious Diseases, University of Pittsburgh School of Medicine 12317 , Pittsburgh, Pennsylvania, USA; 2 Pittsburgh Center for HIV Protein Interactions 129263 , Pittsburgh, Pennsylvania, USA; 3 Department of Cancer Immunology and Virology, Dana-Farber Cancer Institute 1855 , Boston, Massachusetts, USA; 4 Department of Medicine, Harvard Medical School 129263 , Boston, Massachusetts, USA; 5 Division of Infectious Diseases, University of Colorado Anschutz Medical Campus, Aurora, Colorado, USA; Rutgers-Robert Wood Johnson Medical School, Piscataway, New Jersey, USA

**Keywords:** RANBP2, MX2, HIV-1 capsid, cyclophilin

## Abstract

Interlinked interactions between the viral capsid (CA), nucleoporins (Nups), and the antiviral protein myxovirus resistance 2 (MX2/MXB) influence human immunodeficiency virus 1 (HIV-1) nuclear entry and the outcome of infection. Although RANBP2/NUP358 has been repeatedly identified as a critical player in HIV-1 nuclear import and MX2 activity, the mechanism by which RANBP2 facilitates HIV-1 infection is not well understood. To explore the interactions between MX2, the viral CA, and RANBP2, we utilized CRISPR-Cas9 to generate cell lines expressing RANBP2 from its endogenous locus but lacking the C-terminal cyclophilin (Cyp) homology domain and found that both HIV-1 and HIV-2 infections were reduced significantly in RANBP2_ΔCyp_ cells. Importantly, although MX2 still localized to the nuclear pore complex in RANBP2_ΔCyp_ cells, antiviral activity against HIV-1 was decreased. By generating cells expressing specific point mutations in the RANBP2-Cyp domain, we determined that the effect of the RANBP2-Cyp domain on MX2 anti-HIV-1 activity is due to direct interactions between RANBP2 and CA. We further determined that CypA and RANBP2-Cyp have similar effects on HIV-1 integration targeting. Finally, we found that the Nup requirements for HIV infection and MX2 activity were altered in cells lacking the RANBP2-Cyp domain. These findings demonstrate that the RANBP2-Cyp domain affects viral infection and MX2 sensitivity by altering CA-specific interactions with cellular factors that affect nuclear import and integration targeting.

**IMPORTANCE:**

Human immunodeficiency virus 1 (HIV-1) entry into the nucleus is an essential step in viral replication that involves complex interactions between the viral capsid (CA) and multiple cellular proteins, including nucleoporins (Nups) such as RANBP2. Nups also mediate the function of the antiviral protein myxovirus resistance 2 (MX2); however, determining the precise role of Nups in HIV infection has proved challenging due to the complex nature of the nuclear pore complex (NPC) and significant pleiotropic effects elicited by Nup depletion. We have used precise gene editing to assess the role of the cyclophilin domain of RANBP2 in HIV-1 infection and MX2 activity. We find that this domain affects viral infection, nucleoporin requirements, MX2 sensitivity, and integration targeting in a CA-specific manner, providing detailed insights into how RANBP2 contributes to HIV-1 infection.

## INTRODUCTION

Access to the chromosomal DNA contained within the nucleus of target cells is critical for retroviral integration and replication. Among retroviruses, the lentiviruses are uniquely efficient in their ability to enter the nucleus of interphase cells, in which the nuclear membrane is intact. The viral capsid (CA) is the key viral determinant of the ability of human immunodeficiency virus 1 (HIV-1) to infect non-dividing cells (1), and recent research has indicated that the mature CA lattice mimics cellular nuclear transport receptors (NTRs) to mediate high valency interactions with FG-nucleoporins (Nups) that compose the inner channel of the nuclear pore complex (NPC) (2, 3). Other CA-interacting host proteins, including the peptidylprolyl isomerase cyclophilin A (CypA) (4, 5) and the mRNA-processing protein cleavage and polyadenylation specificity factor 6 (CPSF6) (6), can also influence HIV-1 nuclear transport, perhaps via regulating CA-Nup interactions (7, 8). CA-binding host factors implicated in viral nuclear import can furthermore influence sites of HIV-1 integration in the human genome, perhaps by directing preintegration complexes to specific nuclear import pathways, by affecting downstream interactions with CPSF6, which is a key regulator of viral nuclear incursion (9), or through effects on chromatin architecture (10).

**Fig 1:**
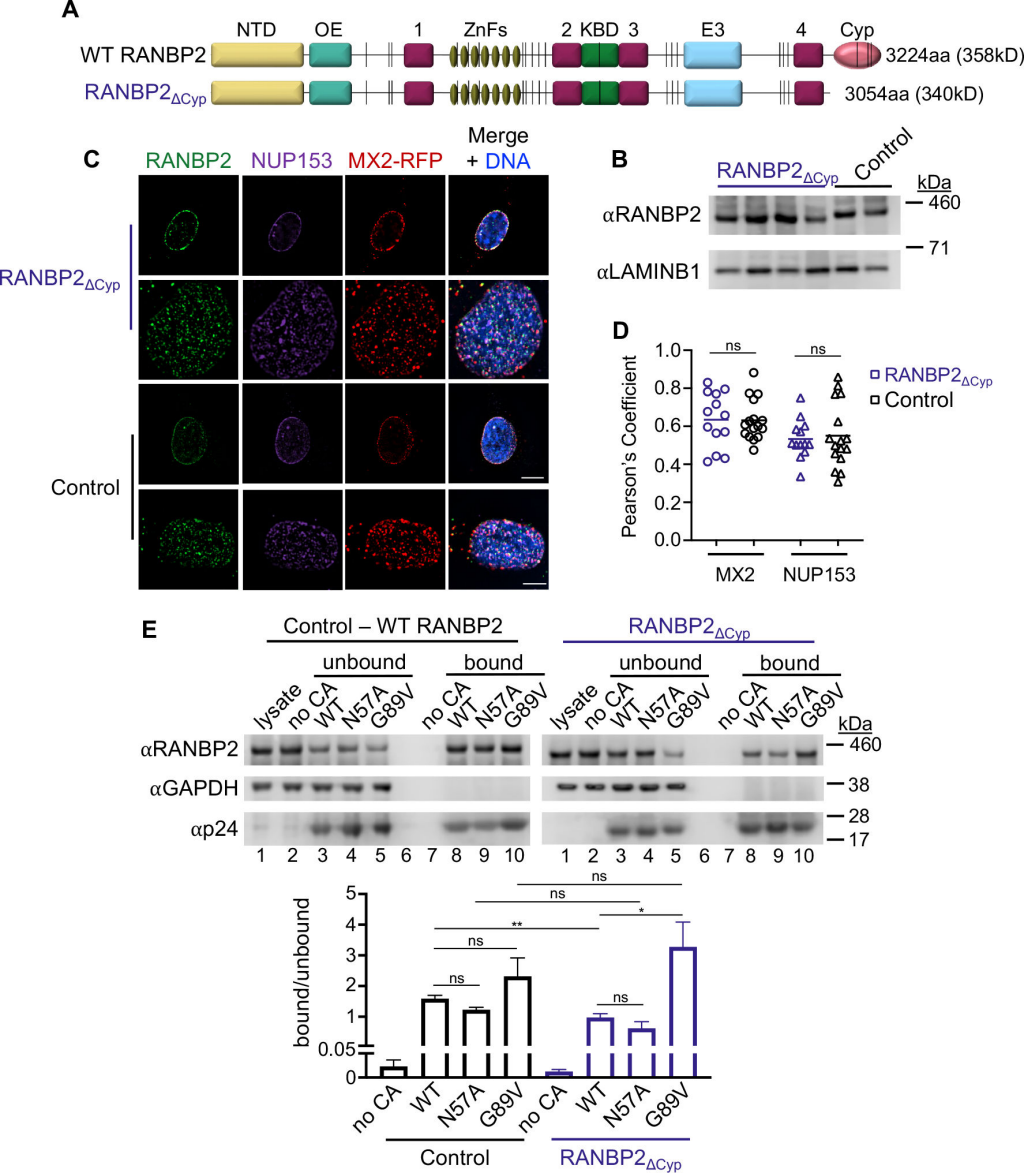
The RANBP2-Cyp domain is not required for MX2 localization to the nuclear envelope or for HIV-1 CA binding. (**A**) Domain architecture of wild-type (WT) RANBP2 and RANBP2_∆Cyp_. NTD (yellow), N-terminal domain; OE (cyan), oligomerization element; 1–4 (magenta), Ran-binding domains; ZnFs (gold), zinc finger motifs; KBD (green), kinesin-binding domain; E3 (blue), SUMO E3 ligase domain; Cyp (pink), cyclophilin homology domain; vertical lines (black), FxFG repeat. (**B**) Western blot analysis of RANBP2 expression in control and mutant cell clones and LAMINB1 loading control. Note the slightly faster mobility of the deletion mutants through the gel. (**C**) Deconvolution microscopic images (single optical sections) of control and RANBP2_∆Cyp_ cells expressing MX2-RFP (red, stably transduced with a doxycycline-inducible vector) were fixed and immunofluorescently stained for RANBP2 (green) and NUP153 (purple) and Hoescht-stained for DNA. (Top) Optical sections are approximately through the center of the vertical dimension on the nucleus, scale bar = 10 µm. (Bottom) Optical sections are approximately coincident with the dorsal surface of the nucleus, scale bar = 5 µm. Representative of nine RANBP2_∆Cyp_ and three control clones. (**D**) Pearson’s coefficient for co-localization of RANBP2 with MX2 (circles) or NUP153 (triangles). Each data point represents an individual cell, and the horizontal bar is the mean (*n* ≥ 13). (**E**) HIV-1 WT, N57A, or G89V CA tubes were assembled *in vitro* and incubated with lysates from control or RANBP2_∆Cyp_ HT1080 cells as indicated. The reaction mixtures were subjected to centrifugation to separate pulldown (bound) fractions from unbound proteins in supernatants. Western blot analysis (top): lane 1—cellular lysates; lane 2—supernatant from control experiments without CA tubes; lanes 3–5—supernatants after incubating cellular lysates with CA tubes; lane 6—empty; lane 7—pulled-down fraction from control experiment in the absence of CA tubes; lanes 7–10—proteins bound to CA tubes. Blot from one RANBP2_∆Cyp_ clone of two tested is shown. Quantification (bottom): ratio of bound to unbound RANBP2 from four replicate blots of control clone and six replicate blots of two RANBP2_∆Cyp_ clones. Statistical significance was determined by one-way analysis of variance (ANOVA) (Kruskal-Wallis test); ns, not significant (*P* ≥ 0.05); *P < 0.05 and **P < 0.01.

RANBP2 (also known as NUP358) is a metazoan-specific component of the cytoplasmic face component of the NPC. The 3224 residue human protein is the largest component of the NPC and is anchored to the cytoplasmic outer ring via its N-terminal domain (NTD) in pentameric bundles connected by interactions between oligomerization elements (OE) (Fig. 1A; [11, 12]). The remaining domains, including several FG repeats, four Ran-binding domains, and a Cyp homology domain, extend into the cytoplasm and are connected by unstructured linker sequences (11, 13). Numerous reports using RNAi-mediated depletion have indicated that RANBP2 plays a major role in nuclear import of HIV-1 and HIV-2 (5, 14–24); however, due to the importance of RANBP2 in multiple cellular processes (25–27) and the numerous pleiotropic effects that can occur upon Nup depletion (18), these results must be interpreted with caution. The C-terminal Cyp domain of RANBP2 contains peptidyl-prolyl isomerase activity, and both genetic and biochemical studies have demonstrated that it directly interacts with HIV-1 CA (5, 15, 21, 22, 28, 29). Furthermore, HIV-1 CA mutant viruses G89V and P90A, which are impaired for CypA binding, exhibit reduced dependence on RANBP2 for infection (5, 18). However, the precise role of the RANBP2-Cyp domain in HIV-1 infection remains controversial. On the one hand, some reports have proposed that CA-RANBP2 interactions are crucial for infection and are a key driver of species-specific primate lentiviral adaptation (21, 29). On the other hand, experiments utilizing ectopic expression of truncated human RANBP2 in mouse cells suggested that direct interactions with the RANBP2-Cyp domain are not required for HIV-1 nuclear import (22).

MX2 is a dynamin-like guanosine triphosphatase whose expression is strongly upregulated by type I interferons (IFNs) and localizes to the NPC via a nuclear localization-like sequence in its first 25 amino acids (30, 31). MX2 inhibits HIV-1 infection prior to chromosomal DNA integration, but after the completion of reverse transcription (30, 32, 33), and current models suggest that it acts by preventing nuclear import of the preintegration complex. HIV-1 CA is the major viral determinant of MX2 sensitivity, and several single-amino acid substitutions in CA have been identified that confer partial or complete resistance to MX2 (30, 32, 34–36). MX2 has also been found to directly bind CA; however, the relevance of this binding for viral inhibition is unclear since MX2-resistant CA proteins are still efficiently bound by MX2 (37, 38). Sensitivity of HIV-1 to MX2 activity is affected by complex interactions between CA and cellular proteins involved in the early stages of HIV-1 infection (reviewed in reference 39), including RANBP2. Knockdown of RANBP2 reduces the antiviral activity of MX2 against HIV-1 in multiple cell types (18, 40). MX2 is also mislocalized upon RANBP2 depletion and exhibits similar Nup requirements for recruitment to the NPC as RANBP2 (18). Additionally, localization of MX2 to the NPC following mitosis occurs subsequent to the recruitment of RANBP2 (18), which is a late event in post-mitotic NPC assembly, and follows the establishment of the structural pore and the central channel (reviewed in references 41, 42), suggesting that MX2 recruitment to the NPC may be dependent on RANBP2. Finally, a yeast two-hybrid screen indicated that RANBP2 may directly interact with the NTD of MX2, although these results were not confirmed via biochemical tests such as co-immunoprecipitation (40). The antiviral activity of MX2 is also affected by HIV-1 CA-CypA interactions, with multiple reports indicating that wild-type HIV-1 (HIV-1_WT_) is only sensitive to MX2 when it is bound by CypA (18, 35, 43, 44). CypA may affect MX2 sensitivity either by affecting viral utilization of cellular nuclear import pathways or by producing an MX2-sensitive CA conformation or state (5, 18, 43).

In this report, we investigated the role of the Cyp domain of RANBP2 in HIV infection and MX2 activity by gene editing of the endogenous *RANBP2* locus. We find that, while abrogation of CA-Cyp domain interactions does not recapitulate the effects of RANBP2 depletion, the Cyp domain is required for efficient HIV infection and integration site selection. We also demonstrate that abrogation of RANBP2-Cyp-CA interactions reduces MX2 activity against HIV-1, but not HIV-2, and that the effects of RANBP2-Cyp on MX2 activity are mediated by direct interactions between HIV-1 CA and the Cyp domain. Finally, we show that deletion of the RANBP2-Cyp domain alters sensitivity of HIV to the depletion of other Nups.

## RESULTS

### Effects of the RANBP2-Cyp domain on MX2 localization and RANBP2-CA interactions

To generate mutant cells endogenously expressing truncated RANBP2 lacking the Cyp domain, we employed a CRISPR/Cas9 adeno-associated virus (AAV)-based gene engineering approach to target the *RANBP2* locus (Fig. S1A). We introduced Cas9 ribonucleoproteins into HT1080 cells that contained guide RNAs targeting exon 27 and exon 29 and an AAV containing donor DNA for homology-directed repair to fuse exons 28 and 29, resulting in the deletion of the C-terminal 170 amino acids of RANBP2 (Fig. 1A; Fig. S1A). Single-cell clones with the desired biallelic mutations were confirmed by western blot (Fig. 1B) and by sequencing of both genomic DNA and mRNA. Since RANBP2 is recruited comparatively late during NPC assembly (reviewed in references 41, 42) and is attached to the cytoplasmic outer-ring spoke via its NTD (11), we would not anticipate deletion of the Cyp domain to alter nuclear envelope localization of RANBP2 or other Nups. As expected, both RANBP2 and NUP153 (which is recruited early in NPC formation (reviewed in references 41, 42) were similarly localized in both control and RANBP2_∆Cyp_ cells. We also found that localization of an MX2-tagRFP fusion to the nuclear envelope was unaffected in RANBP2_∆Cyp_ cells (Fig. 1C and D) indicating that its recruitment to the NPC is independent of the RANBP2-Cyp domain. Additionally, while depletion of RANBP2 resulted in reduced expression of many other Nups (18), we found that deletion of the Cyp domain did not affect the expression of several Nups, including NUP153, ELYS, NUP133, NUP155, NUP88, or NUP62 (Fig. S1B). Collectively, these results suggest that deletion of the Cyp domain does not grossly affect NPC structure. However, since the precise role of this domain in NPC function is unknown, we cannot exclude the possibility that this deletion affects NPC structure/function in ways not detected by the assays that we have used.

We then tested the ability of RANBP2 in control and mutant clones to bind HIV-1 CA *in vitro* (Fig. 1E) using a CA co-pelleting assay. In brief, nanotubes constructed from purified, recombinant CA were incubated with cell extracts. CA-host factor binding was evident via host factor-nanotube co-pelleting following centrifugation (18). We found that both full-length RANBP2 and RANBP2_∆Cyp_ were pelleted with the wild type (WT), FG-binding mutant N57A, and Cyp-binding mutant G89V CAs. The ratio of bound-to-unbound RANBP2 was similar between the WT, N57A, and G89V CA; however, a higher fraction of RANBP2_∆Cyp_ was bound to the G89V than the WT or N57A CA. Additionally, the fraction of bound RANBP2_∆Cyp_ was slightly lower than full-length RANBP2 for the WT, but not for G89V CA. Consistent with the results of an earlier study (17), our results suggest there are both Cyp-domain-dependent and independent interactions between RANBP2 and HIV-1 CA. Because the N57A mutant also bound RANBP2_∆Cyp_, our data indicate that Cyp-domain-independent interactions between RANBP2 and CA are unlikely to be mediated by FG motifs. Such interactions could be mediated by an unknown CA-binding motif in RANBP2 or may reflect an indirect interaction whereby RANBP2 is pelleting with CA via interactions with other host factors.

### Deletion of the RANBP2-Cyp domain affects HIV infection and MX2 sensitivity

**Fig 2:**
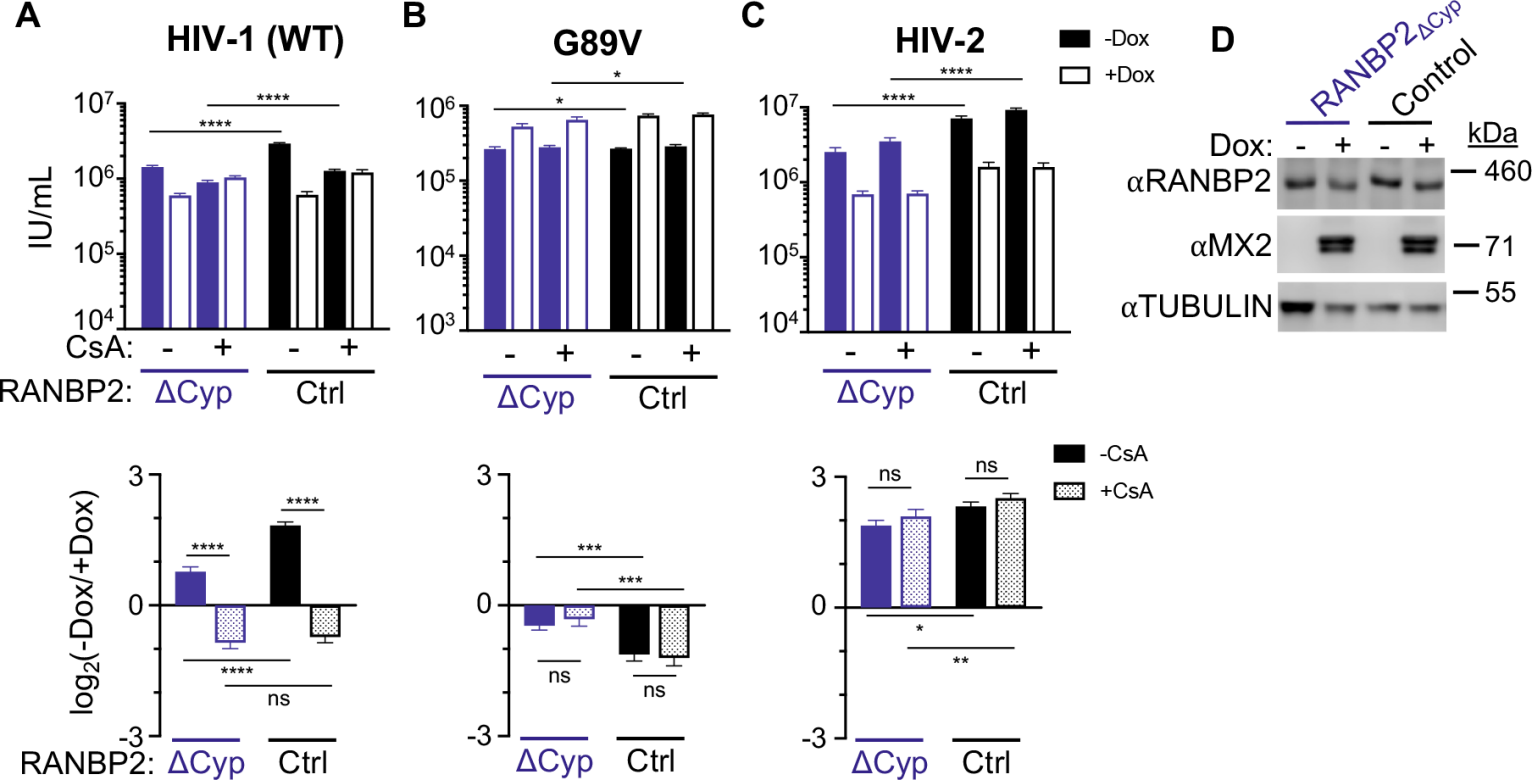
Effects of the RanBP2-Cyp domain on lentiviral infection and MX2 sensitivity. (**A–C**) (Top) Infection of control and RANBP2_∆Cyp_ HT1080 cell clones stably transduced with doxycycline-inducible MX2 in the presence (open bars) or absence (filled bars) of doxycycline and presence or absence of CsA with the indicated GFP reporter viruses. Titers are represented as mean + SEM of infectious units (IU) per mL. RANBP2_∆Cyp_: *n* ≥ 12 technical replicates combined from ≥3 different clones; control: *n* ≥ 8 technical replicates combined from ≥2 different clones; representative of ≥4 independent experiments. Statistical significance was determined by two-way ANOVA (Śídák’s multiple comparisons test). (Bottom) Data from (top) shown as a ratio (fold change of −Dox(−MX2)/+Dox(+MX2)) in the presence (dotted bars) or absence (filled bars) of CsA. Average fold change calculated from three to four technical replicates per experiment; shown is mean + SEM of log_2_(fold change) from four to eight independent experiments. Statistical significance was determined by paired *t* test. (**D**) Western blot analysis of RANBP2, doxycycline-inducible MX2, and tubulin loading control from representative clones. ns, not significant (*P* ≥ 0.05); *P < 0.05, **P < 0.01, ***P < 0.001, and ****P < 0.0001.

To investigate the role of the RANBP2-Cyp domain in HIV-1 infection and MX2 sensitivity, control and RANBP2_∆Cyp_ cell lines were transduced with a lentiviral vector for doxycycline-inducible expression of MX2 and infected with GFP reporter viruses in the presence or absence of the CypA inhibitor cyclosporine A (CsA) at the time of infection (Fig. 2). We observed a modest though significant reduction in HIV-1_WT_ infection (~2.3-fold) as well as a reduction in MX2 sensitivity in RANBP2_∆Cyp_ cells (Fig. 2A), suggesting that in addition to CypA, CA-RANBP2-Cyp interactions affect MX2 activity against HIV-1. However, CsA treatment, which blocks CA-CypA, but not CA-RANBP2-Cyp interactions (5), had similar effects on sensitivity of HIV-1_WT_ to MX2 in both control and RANBP2_∆Cyp_ cells, indicating that RANBP2-Cyp may only affect MX2 activity against CAs that are already bound by CypA. Interestingly, while deletion of RANBP2-Cyp had marginal effects on infection by HIV-1_G89V CA_ as expected, the ability of MX2 to enhance infection of this mutant was reduced in RANBP2_∆Cyp_ cells (Fig. 2B), suggesting there may also be CA-independent effects of the Cyp domain on MX2 activity.

In addition to HIV-1_G89V CA_, we examined the effects of RANBP2-Cyp deletion on several HIV-1 CA mutants with alterations in MX2 and CypA/CsA sensitivity and/or impaired Nup/CPSF6 binding (Fig. S2). Both HIV-1_N57A CA_ and the CPSF6-binding mutant N74D bind Cyps and are affected by CsA treatment in HT1080 cells (5, 44) but have significantly lower affinity for RANBP2-Cyp than HIV-1_WT_ (5). We found that both infectivity and MX2 sensitivity of these CA mutants were unaffected in RANBP2_∆Cyp_ cells (Fig. S2A and B). This is also consistent with previous reports indicating that HIV-1_N57A CA_ and HIV-1_N74D CA_ are less sensitive than HIV-1_WT_ to RANBP2 depletion (5, 20). Collectively, these findings suggest that HIV-1_N57A CA_ and HIV-1_N74D CA_ interact functionally with CypA, but not RANBP2-Cyp. The A92E and G94D mutations appear to stabilize the CypA-binding loop of HIV-1 CA in a conformation resembling the CypA-bound state (45), and these mutants have a complex phenotype, exhibiting CsA resistance or dependence and MX2 resistance that is dependent on the target cell type (reviewed in reference 46). HIV-1_A92E CA_ and HIV-1_G94D CA_ were slightly less sensitive to RANBP2-Cyp deletion than HIV-1_WT_ (~1.3- and ~1.7-fold, respectively), and the sensitivity of HIV-1_G94D CA_, but not HIV-1_A92E CA_ to MX2, was modestly reduced in RANBP2_∆Cyp_ cells (Fig. S2C and D). These findings suggest that stabilization of the CypA-binding loop reduces the dependence on interactions with RANBP2-Cyp. Finally, we tested the effects of RANBP2-Cyp deletion on the T210K CA mutant, which acquired MX2 resistance upon passage in MT4 cells (34) but is sensitive to MX2 in certain contexts, including upon CsA treatment (18, 44). HIV-1_T210K CA_ also has a significant infectivity defect and has altered Nup dependencies, most notably a reduced sensitivity to NUP153 depletion (18), which may be the result of both alterations in cofactor binding as well as an increase in CA stability (47). We found that HIV-1_T210K CA_ was more sensitive than the WT virus to the deletion of RANBP2-Cyp (~3.8-fold), although its sensitivity to MX2 was unaltered (Fig. S2E). The T210K mutation alters the tri-hexamer interface of the CA lattice and affects interactions between the NTD of MX2 and the CA (47); however, our data indicate that in certain contexts, this reduced binding is sufficient to inhibit infection. Collectively, these data indicate that the RANBP2-Cyp domain plays a comparatively minor role in HIV-1 infectivity but affects MX2 sensitivity in a viral CA sequence-dependent manner. While the full antiviral activity of MX2 against HIV-1_WT_ requires both CA-CypA and CA-RANBP2-Cyp interactions, this requirement is highly dependent on the CA sequence, as MX2 sensitivity of several HIV-1 CA mutants is altered by abrogation of interactions with CypA, but not RANBP2-Cyp.

### Distinct effects of RANBP2-Cyp deletion on other lentiviruses

We next explored the requirements for RANBP2-Cyp for infection of other lentiviruses with distinct MX2 sensitivities from HIV-1. While HIV-1 is of chimpanzee origin, HIV-2 (and SIVmac) arose from SIV circulating in sooty mangebeys (reviewed in reference 48). Like HIV-1, infectivity of HIV-2 and SIVmac is reduced upon RANBP2 or NUP153 depletion; however, these viruses have altered interactions with a number of cellular cofactors and distinct requirements for other Nups (18, 21, 49). HIV-2 also has a divergent CypA-binding loop, resulting in lower affinity binding to CypA than HIV-1 (50), and inhibition of HIV-2 by MX2 is independent of CA-CypA interactions (18, 44). HIV-2 is restricted by chimeric TRIM-RANBP2-Cyp (21), indicating that it can interact with RANBP2-Cyp; however, the affinity of this interaction is unknown. We found that HIV-2 was more sensitive to depletion of RANBP2-Cyp than HIV-1 (~4.4-fold infection defect); however, MX2 sensitivity of HIV-2 was only slightly reduced in RANBP2_∆Cyp_ cells (Fig. 2C). SIVmac does not bind Cyps (5, 51, 52) and, accordingly, was not affected by deletion of RANBP2-Cyp (Fig. S3A). SIV from tantalus monkeys (SIVagmTAN) also binds Cyps (53–55), but it is unknown whether it requires RANBP2 for infection. We found that infectivity and MX2 sensitivity of SIVagmTAN were unaffected in RANBP2_∆Cyp_ cells (or by CsA treatment) (Fig. S3B), indicating that, at least in human cells, infectivity of HIV-2, SIVmac, and SIVagmTAN is independent of CA-Cyp interactions. We also investigated how deletion of RANBP2-Cyp affected MX2-resistant non-primate lentiviruses equine infectious anemia virus (EIAV) and feline immunodeficiency virus (FIV) (Fig. S3C and D) (30, 32). EIAV, which does not bind Cyps, was unaffected by RANBP2-Cyp deletion, while FIV infection was slightly elevated (~1.3-fold) in RANBP2_∆Cyp_ cells, in agreement with previous reports indicating that RANBP2 is dispensable for EIAV and FIV infection in human cells (15, 18). These data indicate that while interactions with RANBP2-Cyp are important for HIV-2 infection, they are dispensable for infectivity of multiple other lentiviruses in human cells. However, CA-RANBP2-Cyp interactions may be involved in infection of cells derived from the natural host species of these viruses, especially given that the RANBP2-Cyp domain exhibits evidence of evolution under positive selection (5, 56).

### Direct interactions between RANBP2-Cyp and the HIV-1 CA determine MX2 sensitivity

**Fig 3:**
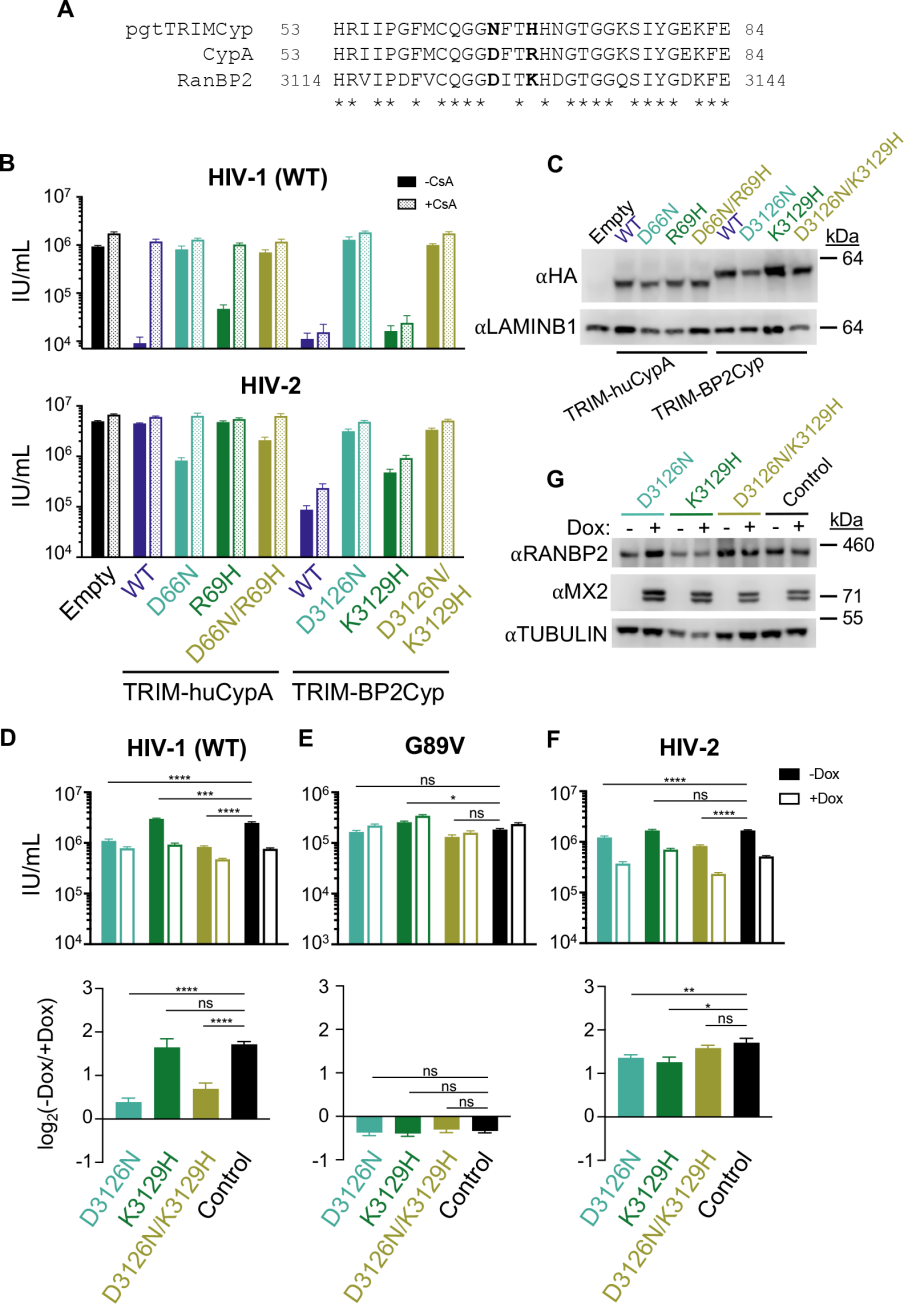
Effect of RANBP2-Cyp point mutations on lentiviral infection and MX2 sensitivity. (**A**) Amino acid alignment of portions of the Cyp domain of pig tailed/rhesus macaque (pgt) TRIMCyp, CypA, and RANBP2-Cyp with key residues involved in CA binding in bold. (**B**) Infectivity of GFP reporter viruses on HeLa cells stably expressing control empty vector, or chimeras of the TRIM5 NTD of owl monkey TRIMCyp with human CypA (huCypA), human CypA_D66N_, CypA_R69H_, CypA_D66N/R69H_ mutants, human RANBP2-Cyp (BP2-Cyp), or BP2-Cyp_D3126N_, BP2-Cyp_K3129H_, BP2-Cyp_D3126N/K3129H_ mutants in the presence (dotted bars) or absence (filled bars) of CsA. Titers are represented as mean + SEM of IU per mL, *n* ≥ 12 technical replicates combined from ≥3 independent experiments. Statistical analysis in Data set S2. (**C**) Western blot analysis of HA-tagged TRIM fusion protein expression and LAMINB1 loading control in stable cell lines in panel B. (**D–F**) (Top) Infection of control and RANBP2 point mutant HT1080 cell clones stably transduced with doxycycline-inducible MX2 in the presence (open bars) or absence (filled bars) of doxycycline with the indicated GFP reporter viruses. Titers are represented as mean + SEM of IU per mL. *n* ≥ 12 technical replicates combined from ≥4 independent experiments. Statistical significance was determined by two-way ANOVA (Śídák’s multiple comparisons test). (Bottom) Data from (top) shown as a ratio (fold change of −Dox(−MX2)/+Dox(+MX2)). Average fold change calculated from three to four technical replicates per experiment; shown is mean + SEM of log_2_(fold change) from four to eight independent experiments. Statistical significance was determined by paired *t* test. (**G**) Western blot analysis of RANBP2, doxycycline-inducible MX2, and tubulin loading control from clones shown in panels D–F. ns, not significant (*P* ≥ 0.05); *P < 0.05, **P < 0.01, ***P < 0.001, and ****P < 0.0001.

To examine the potential role of CA-independent effects of RANBP2-Cyp on HIV-1 infection and MX2 activity, we next generated cells expressing RANBP2 from its endogenous locus but with specific point mutations introduced into the Cyp domain to alter CA-binding specificity. TRIMCyp from both rhesus and pigtailed macaques does not restrict HIV-1 infection and differs from human CypA at only two amino acid positions (D66N and R69H), both of which are outside the active site and differently affect binding to lentiviral CAs (50, 57). We previously demonstrated that CypA_D66N_ and CypA_D66N/R69H_ do not bind HIV-1 CA and that cells expressing these mutant CypA proteins phenocopy CsA-treated and CypA^−/−^ cells with regard to HIV-1 infection and MX2 sensitivity (44). We therefore engineered the corresponding changes of RANBP2 residues D3126 and K3129 (Fig. 3A) and tested these for their effect on HIV CA binding by generated cells expressing HA-tagged chimeric proteins in which the Cyp domain of owl monkey TRIMCyp was replaced with human RANBP2-Cyp (TRIM-BP2Cyp) containing D3126N, K3129H, or both D3126N/K3129H substitutions. TRIM-huCypA fusions with the corresponding D66N, R69H, and D66N/R69H substitutions (44) were included for comparison (Fig. 3B and C). We then challenged these cells with HIV-1_WT_, HIV-2, HIV-1 CA mutants, and other lentiviruses (Fig. 3; Fig. S4). HIV-1_WT_ infection was potently inhibited (~100-fold) by TRIM-BP2Cyp and TRIM-BP2Cyp_K3129H_ and was not rescued by CsA addition as expected, while TRIM-BP2Cyp_D3126N_ and TRIM-BP2Cyp_D3126N/K3129H_ were inactive against HIV-1_WT_. This restriction profile was similar to the corresponding mutations in TRIM-huCypA fusions, suggesting that the same residues are involved in HIV-1 CA binding by CypA and RANBP2-Cyp.

To generate RANBP2 point mutant cell lines, we modified our approach for generating RANBP2_∆Cyp_ cells (Fig. S1C) to express full-length RANBP2 with D3216N and/or K3129H mutations (or WT sequence as a control), followed by a P2A ribosomal skipping sequence and a hygromycin B-resistance gene. Single-cell clones with the desired biallelic mutations were confirmed by sequencing of both genomic DNA and mRNA. As above, RANBP2-Cyp mutant cell lines were then transduced with a lentiviral vector for doxycycline-inducible expression of MX2 and infected with GFP reporter viruses (Fig. 3D through G; Fig. S4). Similar to the phenotype in RANBP2_∆Cyp_ cells, we observed a ~twofold reduction in infection and a reduction in MX2 sensitivity of HIV-1_WT_ in both RANBP2_D3126N_ and RANBP2_D3126N/K3129H_ cells. This effect was specific to mutations that affected binding by RANBP2-Cyp, since both infectivity and MX2 sensitivity of HIV-1_WT_ were similar in control and RANBP2_K3129H_ cells, and suggests that the effects of deletion of the Cyp domain of RANBP2 on HIV-1 infection and MX2 sensitivity are the direct result of abrogating CA-RANBP2-Cyp interactions (although we cannot definitively exclude the possibility that RANBP2_D3126N_ affects interactions with a cellular substrate relevant for HIV-1 CA-MX2 interactions). HIV-1_G89V CA_ was insensitive to all TRIM-Cyp fusions as expected (Fig. S4), and the ability of MX2 to enhance HIV-1_G89V CA_ infection was unaffected in any of the RANBP2 point mutant cell lines (Fig. 3E), further indicating that the reduction in MX2 enhancement of HIV-1_G89V CA_ infection in RANBP2_∆Cyp_ cells (Fig. 2B) is the result of CA-independent effects.

The HIV-1 CA mutants N57A, N74D, and T210K exhibited similar sensitivity to the chimeric TRIM-Cyps as the WT virus (Fig. S4). Accordingly, these CA mutants exhibited similar infectivity and MX2 sensitivity in RANBP2_∆Cyp_ and RANBP2-CA-binding point mutant cell lines (Fig. S2A, B, and E). Notably, HIV-1_A92E CA_ and HIV-1_G94D CA_, which are CsA-dependent in HeLa cells, exhibited a distinct profile of sensitivity to TRIM-BP2Cyp vs TRIM-Cyp fusions (Fig. S4). Additionally, unlike HIV-1_WT_ and other CA mutant viruses, infectivity and MX2 sensitivity of HIV-1_A92E CA_ and HIV-1_G94D CA_ in RANBP2 point mutant cell lines did not appear to correlate with the ability to bind RANBP2-Cyp (Fig. S2C and D), indicating that interactions between these CA mutants and CypA/RANBP2-Cyp are highly complex.

### Requirements for RANBP2-Cyp interactions are virus-specific

The D66N and R69H mutations in rhesus and pigtailed macaque TRIM-Cyp, which eliminate HIV-1 binding, increase binding affinity for HIV-2 CA (50), and HIV-2 therefore has a different sensitivity profile to TRIM-huCypA fusion proteins compared with HIV-1 (Fig. 3B; [(44]). However, HIV-2 exhibited a similar profile as HIV-1 to sensitivity to TRIM-BP2Cyp fusions (Fig. 3B). Similarly, the sensitivity of SIVagmTAN to TRIM-BP2Cyp fusions with D3126N and/or K3129H mutations did not correspond with its sensitivity to TRIM-huCypA fusions with D66N and/or R69H mutations (Fig. S4). These results indicate that, in contrast to the HIV-1 CA, which appears to interact with corresponding residues in CypA and RANBP2-Cyp, CypA and RANBP2-Cyp appear to utilize distinct residues to engage HIV-2 and SIVagmTAN CAs.

While mutation of D3126N abrogated the sensitivity of HIV-2 to TRIM-BP2Cyp restriction, the infectivity of HIV-2 was only slightly reduced in RANBP2_D3126N_ and RANBP2_D3126N/K3129H_ cells (Fig. 3F), suggesting that other residues are responsible for HIV-2 interactions with RANBP2-Cyp. HIV-2 also remained sensitive to MX2 activity in all RANBP2 mutant cells, similar to both SIVmac and SIVagmTAN (Fig. 3F; Fig. S3A and B). These findings indicate that while restriction of HIV-1 by MX2 requires both CA-RANBP2-Cyp and CA-CypA interactions, restriction of HIV-2, SIVmac, and SIVagmTAN is independent of CA-Cyp interactions. Additionally, as expected, EIAV and FIV infection and MX2 sensitivity were largely unaffected in RANBP2 mutant cells (Fig. S3C and D; Fig. S4). Overall, these experiments indicate that direct interactions between RANBP2-Cyp and the viral CA affect infectivity and MX2 sensitivity in a CA sequence-dependent manner.

### HIV-1 integration is mislocalized in RANBP2∆Cyp cells

**Fig 4:**
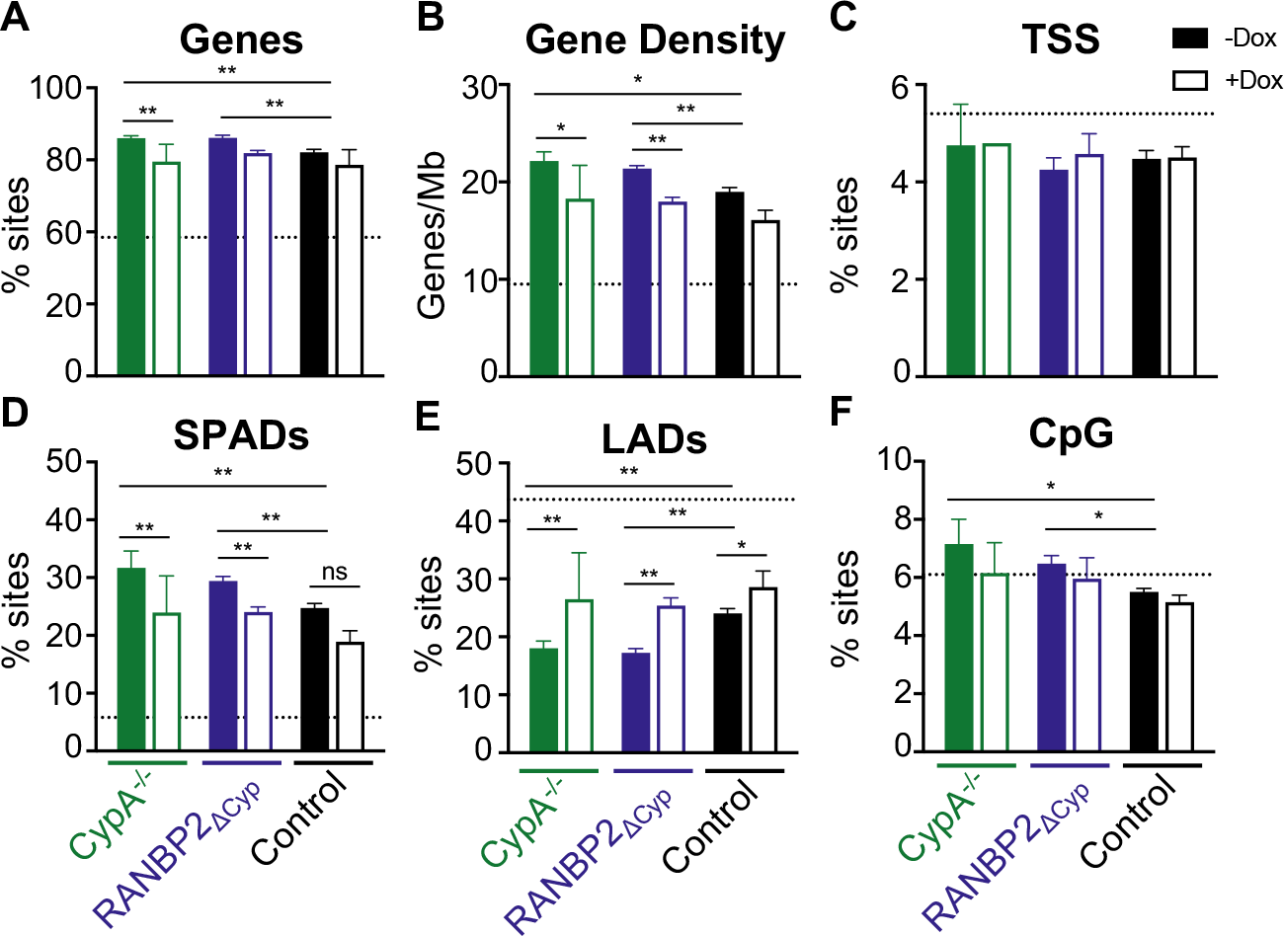
Similar effects of CypA knockout and RANBP2-Cyp deletion on HIV-1 integration site targeting. HIV-1 integration sites in unmutated control, CypA^−/−^, and RANBP2_∆Cyp_ HT1080s stably transduced with doxycycline-inducible MX2 in the presence (open bars) or absence (filled bars) of doxycycline were analyzed for (**A**) genes, (**B**) gene density, (**C**) TSSs, (**D**) SPADs, (**E**) LADs, and (**F**) CpG islands. Dashed lines indicate random integration control values. Infection and integration analyses were performed in duplicate in one representative CypA^−/−^ and control and two RANBP2_∆Cyp_ clones. Control and RANBP2_∆Cyp_ cells were analyzed in two independent experiments. Comparisons between infection conditions were analyzed by Fisher’s exact test (**A, C–F**) or Wilcoxon rank-sum test (**B**). (*P* ≥ 0.05), *P < 0.05, and **P < 0.0001.

HIV-1 integration, which favors active transcription units within gene-dense regions and speckle-associated domains (SPADs) and disfavors heterochromatin regions such as lamina-associated domains (LADs), is mediated by interactions between HIV-1 integrase and CA with multiple host cell proteins (reviewed in references 10, 58, 59). Previous reports have indicated opposing effects of RANBP2 and CypA on HIV-1 integration in HeLa cells, with RANBP2 knockdown leading to reduced integration in gene-dense regions and CpG islands (23) and CsA treatment (or infection with G89V/P90A CA mutants) leading to increased integration in gene-dense regions, CpG islands, and SPADs and a decrease in integration in LADs (5, 60). MX2 has also been shown to affect integration patterns in HOS cells, slightly reducing genic integration, and integration surrounding transcriptional start sites (TSSs) and CpG islands (33). To determine how the RANBP2-Cyp domain specifically affects HIV-1 integration targeting, we assessed integration in CypA-deficient (44), RANBP2_∆Cyp_, and control cells in the presence or absence of MX2 expression (Fig. 4 and Data set S1). Cells were infected with WT HIV-1 containing a heterologous sequence embedded in the U3 region to enable discrimination from the lentiviral vector used for doxycycline-inducible MX2 expression (61). Proviral integration sites were amplified, sequenced, and analyzed for association with genes, gene density, SPADs, LADs, TSSs, and CpG islands as previously described (62, 63). We found that integration into genes was increased in both CypA^−/−^ and RANBP2_∆Cyp_ cells (Fig. 4A), as was integration into gene-dense regions (>20 genes/Mb) (Fig. 4B), SPADs (Fig. 4D), and CpG islands (Fig. 4F), while there were fewer integration sites in LAD-associated DNA (Fig. 4E) and no change in integration within TSSs (Fig. 4C). MX2 expression resulted in reduced targeting to gene-dense regions and SPADs, with a concomitant increase in integration in LADs (although the effects were not statistically significant for gene density/SPADs in control cells, there was a clear trend). Notably, although the ability of MX2 to restrict HIV-1 infection is reduced in CypA^−/−^ and RANBP2_∆Cyp_ cells (44) (Fig. 2), we found that the effect of MX2 on integration targeting was maintained in the absence of CA-Cyp interactions (Fig. 4B, D, and E). These findings suggest that while CA-Cyp interactions are required for MX2-mediated restriction, MX2 also affects the interactions of HIV-1 CA with other cellular factors involved in integration targeting (e.g., Nups and CPSF6) and/or the kinetics of nuclear import (or CA uncoating) independently of CA-Cyp interactions.

### Deletion of the RANBP2-Cyp domain alters Nup requirements for HIV-1 infection and MX2 sensitivity

**Fig 5:**
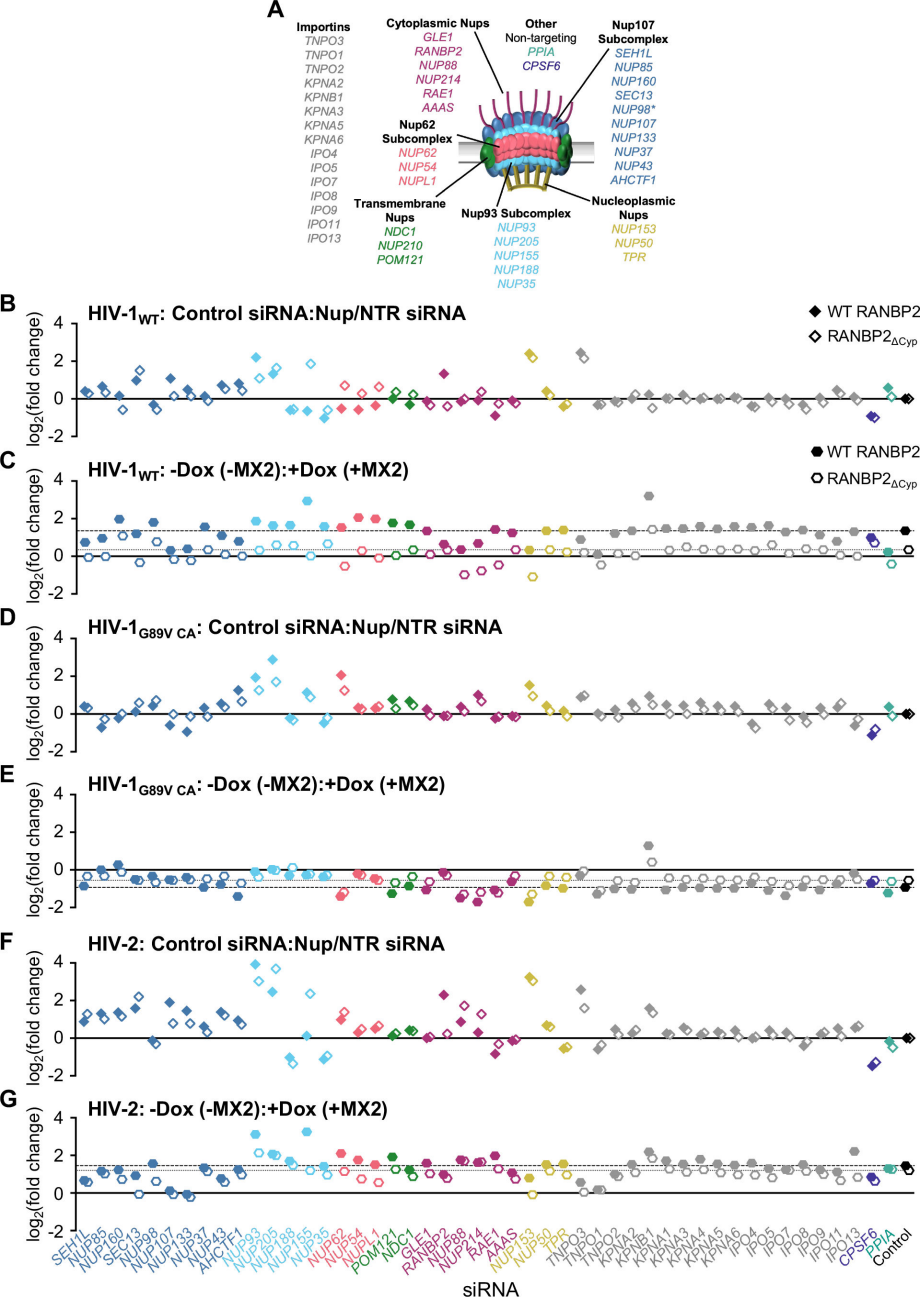
Effect of RANBP2-Cyp on the Nup requirements for HIV infection and MX2 sensitivity. (**A**) Schematic representation of the NPC and genes included in siRNA library color coded by subcomplex. (*NUP98 is listed as a member of Nup107 subcomplex, however. Nup98 and Nup96 are produced following autoproteolytic cleavage of a polyprotein precursor (64, 65); this siRNA will target both Nups). Importins/nuclear transport receptors (NTRs) included in the siRNA library listed in gray. Also included, siRNA targeting CypA, CPSF6, and a non-targeting control siRNA. Effect of Nup/NTR depletion on (**B**) HIV-1_WT_, (**D**) HIV-1_G89V CA_, and (**F**) HIV-2 infection in control (filled diamonds) and RANBP2_∆Cyp_ (open diamonds) cells shown as a ratio (log_2_[fold change of control siRNA/Nup/NTR siRNA]). Average fold change calculated from eight technical replicates from two independent experiments. (**C, E, G**) Effect of Nup/NTR depletion on MX2 sensitivity of (**C**) HIV-1_WT_, (**E**) HIV-1_G89V CA_, and (**G**) HIV-2 in control (filled hexagons) and RANBP2_∆Cyp_ (open hexagons) cells shown as a ratio (log_2_[fold change of −Dox(−MX2)/+Dox(+MX2)]). Dashed line represents effect on control cells; dotted line represents effect on RANBP2_∆Cyp_ cells; siRNAs are color coded by subcomplex as in panel A.

Our previous work demonstrated that multiple Nups functionally interact in complex ways that affect the efficiency of HIV infection and the antiviral activity of MX2 (18). Therefore, we next investigated whether the RANBP2-Cyp domain affects the requirements for other Nups using our previously validated siRNA library targeting Nups and NTRs (Fig. 5A; Fig. S5) (18). Despite the previously discussed complications of Nup depletion, this approach remains valuable for the identification of changes in viral dependence upon Nups under various conditions (e.g., comparison of WT versus CA mutant viruses or WT versus mutant cell lines) and can provide valuable insights for more in-depth future investigation of individual Nups. Control and RANBP2_∆Cyp_ cells were transfected with siRNA before being split into replicate wells for MX2 induction (via doxycycline addition) followed by infection with HIV-1_WT_ (Fig. 5B and C; Fig. S5; Fig. S6A and B). Deletion of the RANBP2-Cyp domain did not result in global changes to HIV-1_WT_ sensitivity to Nup depletion, as most Nup and NTR depletions had similar effects on HIV-1_WT_ infection in both RANBP2_WT_ and RANBP2_∆Cyp_ cells (Fig. 5B). There were, however, a number of differences in Nup requirements for HIV-1_WT_ infection in RANBP2_∆Cyp_ cells, including a dramatic increase in sensitivity to NUP155 knockdown, and decreased sensitivity to NUP107, NUP93, and RANBP2 depletion. Additionally, while Nup62 subcomplex depletion modestly enhanced HIV-1_WT_ infection in control cells, depletion of members of this subcomplex slightly reduced infectivity in RANBP2_∆Cyp_ cells. Many Nup/NTR depletions also had similar effects on MX2 activity against HIV-1_WT_ in both control and RANBP2_∆Cyp_ cells (Fig. 5C) (e.g., increased upon KPNB1 depletion and decreased upon SEH1, NUP107, NUP133, ELYS, and TNPO1 depletion). However, there were many Nup depletions that altered the sensitivity of HIV-1_WT_ to MX2 in RANBP2_∆Cyp_ cells, most notably a total loss in MX2 sensitivity upon NUP155 depletion, which had enhanced antiviral activity in WT cells.

As expected, sensitivity of HIV-1_G89V CA_ to Nup depletion was also largely unaffected by RANBP2-Cyp deletion (Fig. 5D; Fig. S6C and D), although we did observe some changes in sensitivity to NUP107, NUP133, NUP93, NUP205, and NUP62 depletion, indicating that the Cyp domain may affect the function/conformation of other Nups. Similarly, MX2-mediated enhancement of HIV-1_G89V CA_ infectivity was amplified and diminished by similar Nup depletions in both control and RANBP2_∆Cyp_ cells (Fig. 5E) (e.g., amplified by NUP62, NUP88, NUP214, RAE1, and NUP153 depletion and diminished by NUP85, NUP160, Nup93 subcomplex, NUP54, RANBP2, and TNPO3 depletion). Notably, while the antiviral activity of MX2 against HIV-1_WT_ was reduced in control cells upon depletion of the cytoplasmic ring Nups, NUP88, NUP214, and RAE1 and upon depletion of NUP153, MX2 expression enhanced HIV-1_WT_ infection upon these knockdowns in RANBP2_∆Cyp_ cells, similar to its effect on HIV-1_G89V CA_ in both control and RANBP2_∆Cyp_ cells (Fig. 5B through E). MX2 also modestly enhanced HIV-1_WT_ infection upon NUP62, RAE1, and TNPO1 knockdown. These results suggest that the amplification of MX2-dependent increases in HIV-1_G89V CA_ infectivity upon several Nup depletions results from a failure of this mutant to interact with RANBP2-Cyp and may therefore be independent from the effects of CypA on nuclear import and MX2 sensitivity.

Finally, we investigated the sensitivity of HIV-2 to Nup/NTR depletion in RANBP2_∆Cyp_ cells (Fig. 5F and G; Fig. S6E and F). As was the case for HIV-1, most knockdowns had similar effects on HIV-2 infection in control and RANBP2_∆Cyp_ cells. Like HIV-1, sensitivity of HIV-2 to NUP155 depletion was dramatically increased in RANBP2_∆Cyp_ cells, as was elimination of sensitivity to RANBP2 depletion (Fig. 5B and F). We also observed some HIV-2-specific effects of Nup/NTR depletion (e.g., increased sensitivity to NUP205, NUP88, and NUP214 knockdown and decreased sensitivity to TNPO3 knockdown). HIV-2 sensitivity to MX2 was retained in RANBP2_∆Cyp_ cells (Fig. 2A and B) and while some Nup depletions reduced the susceptibility of HIV-2 to MX2 in RANBP2_∆Cyp_ cells (e.g., SEC13, NUP98, NUP93, NUP155, the Nup62 subcomplex, and NUP153) (Fig. 5G), we did not observe any instances in which MX2 enhanced HIV-2 infectivity of RANBP2_∆Cyp_ cells, as it did for HIV-1. Collectively, these findings indicate that deletion of the RanBP2-Cyp domain alters the nuclear import pathways available to HIV (or nuclear import kinetics) predominantly via its effects on CA-specific interactions with other NPC components and further suggests that alterations in MX2 sensitivity are determined by these Cyp domain-dependent changes in nuclear import pathway usage, kinetics, and/or CA conformation.

## DISCUSSION

Interactions with numerous host proteins are required for retroviral access to the nucleus and for integration. Nups appear to play a key role in both HIV-1 passage across the nuclear envelope and downstream interactions that affect viral access to the host chromatin. Complexities in NPC composition have made determining the functionally relevant interactions between HIV-1 CA and Nups a significant challenge. This difficulty extends to our understanding of MX2 activity, which is clearly affected by complex interactions with its cellular environment. Our previous work that employed a systematic knockdown approach indicated that multiple Nups are involved in both HIV-1 infection and the antiviral activity of MX2 (18). However, data obtained using knockdown/knockout approaches must be interpreted with caution due to the existing variability in NPC composition, the significant pleiotropic (or off-target) effects of many Nup depletions, and the potentially confounding effects of incomplete knockdown (18). Similarly, studies establishing direct interactions between Nups and CA either by demonstrating susceptibility to chimeric TRIM5-fusion proteins or by *in vitro* biochemical approaches may or may not indicate that such interactions have functional consequences in the context of virus infection. Modern gene-editing technology now provides the opportunity to directly test the role of individual Nup domains in HIV-1 infection and MX2 activity by generating cells expressing Nups from their endogenous loci, but with specific domains deleted. This precise construction of deletion mutants allows for the modification of the functional capabilities of the NPC without entirely disrupting its assembly or altering overall expression levels. Using this approach, we investigated the function of the Cyp homology domain of the cytoplasmic filament Nup RANBP2 in HIV-1 infection and MX2 activity. This deletion did not appear to affect expression of other Nups or the recruitment of NUP153, RANBP2, or MX2 to the NPC; however, the extent of the effects of this deletion on cellular nucleocytoplasmic trafficking remain unknown. We demonstrated that HIV-1 CA-Cyp domain interactions have small effects on the HIV-1 titer in single-cycle infection assays and affect integration site targeting and MX2 activity in a manner similar to CA-CypA interactions (Fig. 2A and B; Fig. 3D and E; Fig. 4). We also determined that the RANBP2-Cyp domain affects the requirements for other Nups for infection and MX2 sensitivity (Fig. 5).

The antiviral activity of MX2 against HIV-1 is reduced in cells containing mutations in CypA that affect CA recognition (44), and in this report, we demonstrate that similar mutations in RANBP2-Cyp reduced the antiviral activity of MX2. Importantly, however, the effect of MX2 on integration site targeting was retained in both CypA^−/−^ and RANBP2_∆Cyp_ cells (Fig. 4). A recent report has proposed that MX2 restricts HIV-1 (and herpes simplex virus-1) by forming biomolecular condensates that act as a nuclear pore decoy (66); the authors further suggest that MX2-resistance of the P90A CA mutant is the result of its lower propensity to accumulate within these condensates. Our findings reported here would indicate that HIV-1 CA interacts with MX2 regardless of its conformation/interaction with other cellular cofactors, whether this interaction results in abortive infection or altered integration targeting is determined (at least in part) by CA-Cyp interactions. This is also consistent with our previous work demonstrating that MX2-resistance is context dependent and is affected by CA mutations, cell type, cell cycle, and CypA (18, 44) and that MX2 can increase infection of HIV-1 in some contexts (Fig. 5) (18).

Knockdown of RANBP2 reduced HIV-1 integration into gene-dense regions, similar to cells lacking CPSF6 (or CA mutants that do not bind CPSF6) (23, 63, 67, 68); however, we demonstrate here that this effect is not mediated by CA-RANBP2-Cyp interactions, as integration was mislocalized in RANBP2_∆Cyp_ cells in a manner similar to CypA^−/−^ cells (increased targeting to gene-dense regions and increased SPAD targeting) (Fig. 4). Given the increasing evidence that CypA is likely stripped from the CA at the nuclear pore (69, 70) and that CypA-CA interactions affect nuclear entry and integration in a CPSF6-dependent manner (60), one possibility is that sequential binding of CA to CypA, followed by RANBP2-Cyp, affects downstream interactions with CPSF6. While CPSF6 does not appear to play a major role in MX2 activity (Fig. 5) (18), it has been proposed that CPSF6 and MX2 cooperate to prevent interactions with RANBP2 (71).

Deletion of RANBP2-Cyp abrogated the sensitivity of HIV-1_WT_ and HIV-2 to RANBP2 knockdown, since there was no further reduction in infectivity upon RANBP2 depletion in RANBP2_∆Cyp_ cells, similar to the phenotype observed for Cyp-binding mutant CA viruses in cells expressing WT RANBP2 (30, 32, 34–36) (Fig. 5). Interestingly, CsA treatment also rescues HIV-1 infection from RANBP2 (and NUP153) knockdown in some cell types (5, 18, 72), suggesting that CA interactions with both CypA and the RANBP2-Cyp domain determine the requirement for RANBP2. Furthermore, the effects of RANBP2 knockdown were only slightly larger than the effects of Cyp domain deletion on HIV-1 and HIV-2 infection which were similar (2.5- vs 2.3-fold and 4.9- vs 4.4-fold, respectively). Therefore, while *in vitro* experiments indicate that additional domains of RANBP2 can interact with the HIV CA (3, 17) (Fig. 1E), our data suggest that these interactions are not required for infection, at least in HT1080 cells.

RANBP2-Cyp-CA interactions also appear to determine the requirement for NUP155, as HIV-1_G89V CA_ was sensitive to NUP155 depletion, and both HIV-1_WT_ and HIV-2 became highly sensitive to NUP155 knockdown in RANBP2_∆Cyp_ cells (Fig. 5). NUP155 is an inner-ring Nup that lacks an FG domain (73) and does not bind HIV-1 CA tubes *in vitro* (18). It will therefore be of interest in future investigations to determine how NUP155 affects HIV-1 and MX2 without directly interacting with CA (perhaps, by affecting the structural conformation of individual nuclear pores [74, 75]).

One interpretation of our findings is that elimination of CA-RANBP2-Cyp interactions results in utilization of alternative, less efficient nuclear import pathways (e.g., NPCs with different composition or conformation), which thereby alters both MX2 sensitivity and integration site targeting. An alternative, but not mutually exclusive, explanation is that these manipulations affect the kinetics and/or location of CA uncoating/release of viral DNA. This interpretation is supported by the recent findings that the HIV CA acts as its own karyopherin to facilitate interactions with the FG mesh of the NPC (2, 3), which would suggest that alterations in the NPC that affect, but do not eliminate interactions between CA and FGs, would result in only small defects in infectivity as measured by GFP reporter expression, as we observed in this investigation. Additionally, the observation that MX2 affects HIV-1 integration site targeting even when infectivity is not affected (Fig. 4) supports a model in which MX2 affects the timing and/or location of loss of core stability. We would further expect that the comparatively small defects in infectivity observed upon RANBP2-Cyp deletion would likely have more dramatic consequences in cells in which the virus is in danger of improper viral DNA sensing, such as in macrophages, which cannot currently be effectively manipulated using the approaches employed here. An additional limitation of the current study is that while endogenous MX2 expression is upregulated by type I IFN stimulation, we utilized ectopic expression of MX2 to isolate the effects of MX2 from those of other ISGs. Therefore, future investigations concerning the role of the RANBP2-Cyp domain in MX2 sensitivity in the context of IFN stimulation, in cells capable of supporting viral replication and in those lacking additional portions of the cytoplasmic extensions of RANBP2, will be important to determine crucial interactions between RANBP2, MX2, and HIV-1 that mitigate the outcome of infection.

In summary, this report demonstrates a specific role for the Cyp domain of RANBP2 in HIV infection and MX2 sensitivity and further indicates that precise manipulation of NPCs is key to revealing the functional interactions that determine the outcome of HIV-1 infection. Furthermore, this approach should be of great value in revealing the details of normal cellular nucleocytoplasmic trafficking.

## MATERIALS AND METHODS

### Plasmid construction

#### TRIM5-fusions

An LHCX MLV vector (Clontech) was engineered to express the NTDs (RING, B-Box and coiled coil) from owl monkey (omk) TRIMCyp followed by an HA tag and cloning sites (*NotI*/*SalI*) that allowed introduction of various proteins as described in references 76, 77. TRIM-huCypA fusions were previously reported (44). Plasmids expressing TRIM5-HA N-terminus fused to the human RANBP2-Cyp domain or the human RANBP2-Cyp domain with the D3126N, K3129H, or D3126N/K31299H changes were generated by overlap PCR with the primers indicated in Table S1.

#### AAV donors

To construct the pAAV-*RANBP2* donor plasmid for the generation of RANBP2_∆Cyp_ cells (Fig. S1A), a 1,965 bp region of the *RANBP2* locus lacking the Cyp domain and intron 28 (bases 62242 to 63214 and 64454 to 65446 NCBI Reference Sequence: NG_012210.2) was synthesized as a gBlock fragment (IDT) and inserted into the pAAV packaging plasmid (Cell Bio Labs) using *Nhe*I and *Xho*I. The PAM site for crRNA 1 was mutated from TGG to TGA and for crRNA to from GGG to GCC. For pAAV-*RANBP2* donor plasmids for RANBP2 point mutant cell lines, a gBlock fragment was synthesized containing an 890 bp region of the *RANBP2* locus lacking intron 28 (bases 62831 to 63417 and 64151 to 64453) followed by a P2A cleavage site and hygromycin resistance gene, and 57 bases of exon 29 of *RANBP2* following the stop codon (bases 64454 to 64510) was inserted into pAAV-RANBP2_∆Cyp_ using *BamHI* and *HindIII*. A silent *KpnI* site was introduced into intron 27 as shown in Fig. S1B. The WT donor template was synthesized; then, RANBP2_D3126N_, RANBP2_K3129H_, and RANBP2_D3126N/K3129H_ donors were generated via overlap PCR with the primers indicated in S1 table and inserted into pAAV-RANBP2-2A-Hygro using *KpnI* and *NdeI*.

### Viruses

All viruses were generated by transfection of HEK293T cells using polyethyleneimine (PolySciences). GFP reporter proviral plasmids HIV-1_NL4-3_ΔEnv-GFP (HIV-1, HIV-1_G89V_, HIV-1_A92E_, and G94D CA mutants [78]), NHGCapNLNM (WT, HIV-1_N57S_, HIV-1_N57A_, HIV-1_N57D_, HIV-1_N74D_, and HIV-1_T210K_ CA mutants [18, 79]), HIV-2_ROD_ΔEnv-GFP, SIV_MAC_ΔEnv-GFP, SIV_AGM_TANΔEnv-GFP (80), and HIV-1_NL4-3_ΔEnv-Luc-U3tag (61) (10 µg) were co-transfected with 1 µg of VSV-G expression plasmid. For EIAV and FIV, three plasmid vector systems (81, 82) were used to generate GFP reporter viruses, whereby 5 µg of Gag-Pol, 5 µg of packageable genome, and 1 µg of VSV-G expression plasmids were co-transfected.

### Infection assays

Infectivity was measured in HeLa or HT1080 cells seeded in 96-well plates at 5 × 10^3^ cells per well and inoculated with serial dilutions of VSV-G-pseudotyped GFP reporter viruses in the presence of 4 µg mL^−1^ polybrene (Sigma-Aldrich). Where indicated, CsA (Sigma-Aldrich) was added to the cultures at the time of infection at 5 µM. Infected cells (%GFP positive of viable cells) were enumerated by FACS analysis using an Attune NxT coupled to an Autosampler (Invitrogen).

### Cell lines

Culture of cell lines has been previously described (18, 44). HEK293T, HeLa, and HT1080 cell lines were maintained in Dulbecco’s modified Eagles medium (DMEM, Gibco) with 10% fetal calf serum (Gibco), gentamicin (Gibco), and Plasmocin prophylactic (InvivoGen). Plasmocin was not present in cultures during infection, transduction, or transfection. Cells were purchased from ATCC and were assumed to be authenticated by their supplier and were not further characterized. Cells were monitored quarterly for retroviral contamination by SYBR-Green-based PCR RT assay (83, 84) and tested for mycoplasma contamination by MycoStrip detection kit (InvivoGen). Derivatives of HeLa and HT1080 cells containing doxycycline-inducible MX2 or fusion proteins were generated by transduction with LKO-derived lentiviral vectors (34) followed by selection in 1 µg mL^−1^ puromycin (Sigma-Aldrich). HeLa cells stably expressing TRIM fusions were generated by transduction with LHCX-derived vectors followed by selection in 100 µg mL^−1^ hygromycin B (Gibco). Vector stocks for transduction were generated by co-transfection of HEK293T cells with a VSV-G expression plasmid, an HIV-1_NL4-3_ Gag-Pol expression plasmid, and LKO-derived vector or an MLV Gag-Pol expression plasmid and an LHCX-derived vector using polyethyleneimine (PolySciences). Expression was induced in pLKO-transduced cell lines through an overnight treatment with 500 ng/mL doxycycline hyclate (Sigma-Aldrich) prior to challenge with retroviruses or retroviral vectors.

### Generation of RANBP2_∆Cyp_ and point mutant cell lines

RANBP2 mutant cell lines were generated via CRISPR/Cas9-mediated genome editing using AAVs for delivery of donor sequences for homology-directed repair as previously described (44). AAVs containing donor sequence for homology-directed repair were generated by co-transfection of HEK293T cells with pAAV-*RANBP2*, pAAV-RC (DJ), and pHelper (CellBio Labs) at a 1:1:1 ratio using polyethyleneimine (PolySciences). Supernatant was collected 72 h post-transfection. 1 × 10^5^ HT1080 cells in a 24-well plate were infected with 200 µL of AAV-containing supernatant 4–6 h prior to reverse transfection with Cas9 ribonucleoprotein complexes (RNPs). Custom Alt-R CRISPR Cas9 guide RNAs targeting the *RANBP2* locus were designed using the IDT website. RNPs containing crRNA 1 or 2:tracerRNA duplexes were generated according to manufacturer instructions: 1 µL of 100 µM crRNA and 1 µL of 100 µM tracrRNA-ATTO 550 (IDT) in 98 µL of Nuclease-Free Duplex Buffer (IDT) were heated at 98°C for 5 min and then cooled to room temperature. Fifteen microliters of annealed guide RNA oligos were mixed with 15 µL of 1 µM recombinant Cas9 (IDT) in 220 µL of Opti-MEM media (Gibco) and incubated for 5 min at room temperature; complexes were used immediately or stored at −80°C. HT1080 cells were reverse transfected in 96-well plates with RNPs using RNAiMax (Thermo Fisher) according to manufacturer instructions; transfection complexes containing 15 µL of each RNP was mixed with 1.2 µL of RNAiMax and 18.8 µL of OptiMEM for 20 min at room temperature, followed by addition of 4 × 10^4^ cells/well. For generation of point mutant cell lines, 1 µM Alt-R HDR Enhancer V2 (IDT) was included. RANBP2_∆Cyp_ control cells were transfected with RNPs containing the IDT negative control crRNA. Sixteen hours post-transfection, cells were transferred to a 24-well plate, and AAV infection/transduction was repeated after an additional 24–48 h. Single-cell clones were then derived by limiting dilution. For point mutant and WT control cell lines, bulk populations were placed in Hygromycin selection for 5 days prior to seeding clones.

RANBP2_∆Cyp_ clones were screened via PCR amplification from genomic DNA using BP2delCyp Check HR F and R primers (see Table S1). RANBP2 point mutant clones were screened via PCR amplification from genomic DNA using BP2 Exon 26 F1 and BP2 Cyp R followed by *KpnI* digestion. Candidates were confirmed by PCR and sequencing from both genomic DNA and cDNA (using SuperScript III Reverse Transcriptase, Thermo Fisher). Genomic DNA and mRNA were extracted using NucleoSpin Tissue and NucleoSpin RNA kits (Macherey-Nagel), respectively. Candidates were confirmed by western blot and sequencing from genomic DNA. Nine RANBP2_∆Cyp_ and three control clones were selected for experiments; each experiment included three to four RANBP2_∆Cyp_ and two to three control clones. Two homozygous RANBP2_WT-2A-Hygro_, two RANBP2_D3126N_, three RANBP2_D3126N/K3129H_, and one RANBP2_K3126H_ clones were identified and selected for experiments. Experiments show data from multiple clones combined or one representative clone (as we previously reported, HT1080 cells exhibit limited clonal variability [44]).

### CA-binding assay with HIV-1 CA tubes

CA(WT), CA(G89V), and CA(N57A) proteins were expressed from pET3a in BL21-DE3 cells and purified as previously described (85–87). CA nanotubes were assembled in a high-ionic strength buffer (15 mM Tris-HCl, pH 8.0; 2 M NaCl) as described (88, 89). Binding assays with HIV-1 CA nanotubes were performed as described (18). In brief, control and RANBP2_∆Cyp_ HT1080 cells were lysed by adding a passive lysis buffer (Promega) supplemented with protease inhibitor cocktail (Roche). NaCl concentration in lysates was adjusted to 2 M, and lysates were centrifuged at 13,000 × g for 2 min at 4°C. Then, the supernatant of the cell lysates was added to the preformed CA nanotubes and incubated at room temperature for 30 min. Following centrifugation at 13,000 × g for 2 min at 4°C , the supernatant was saved, and the pellet was washed three times with the high-ionic strength buffer. LDS Reducing Sample buffer (Thermo Scientific Chemicals) was added to both pulled down and unbound fractions, and they were subjected to SDS-PAGE. The proteins of interest were detected by immunoblotting using respective antibodies.

### RNA interference

RNA interference using a custom siRNA library (Table S2) targeting Nups and NTRs was performed as previously described (18). RANBP2_∆Cyp_ or control HT1080 cells stably transduced with doxycycline-inducible MX2 were reverse transfected with 25 pmol of siRNA (SMARTpool, Dharmacon; Table S2) using Lipofectamine RNAiMax (Invitrogen) at a concentration of 5 × 10^4^ cells/mL in 12-well plates. Non-targeting siRNA were used as controls; no significant difference in viral infectivity or Mx2 restriction was observed for each of these controls; as such, for each experiment, only the non-targeting siRNA control is shown. Twenty-four hours after transfection, cells were trypsinized, diluted 1:2.5 and re-plated in 96-well plates, and treated with doxycycline, followed by infection with GFP reporter viruses 36–48 h later (See Fig. S5).

### Western blotting

Cell suspensions were lysed in NuPage LDS sample buffer (Invitrogen), followed by sonication, and separated by electrophoresis on NuPage 4%–12% Bis-Tris gels or 4%–8% Tris-Acetate gels (Invitrogen) and blotted onto polyvinylidene fluoride (BioRad Laboratories). Membranes were incubated with the antibodies listed in Table S3, followed by incubation with goat anti-rabbit-HRP or goat anti-mouse-HRP secondary antibodies (Jackson ImmunoResearch). SeeBlue Plus2 and HiMark Pre-stained Protein Standards (Thermo Fisher) were used. Blots were developed with SuperSignal West Femto Maximum Sensitivity Substrate (Thermo Fisher) and imaged on a C-Digit scanner (LI-COR Biosciences).

### Immunofluorescence

RANBP2_∆Cyp_ or control HT1080 cells stably transduced with doxycycline-inducible MX2-tagRFP were seeded onto an eight-chamber gelatin-coated glass coverslip (Ibidi) and treated with 500 ng/mL doxycycline 16 h prior to fixation with 4% paraformaldehyde. Cells were then permeabilized with 0.5%Triton X-100 (Thermo Scientific) and immunostained with the indicated antibodies (Table S3) followed by goat anti-mouse or goat anti-rabbit Alexa 488 or Alexa 647 secondary antibodies (Molecular Probes). DNA was stained with Hoescht 33342 (Thermo Scientific). Cells were visualized on an EVOS M7000 digital microscope (Thermo Scientific) at 100× magnification. Image generation, deconvolution analysis, and co-localization measurements were completed with the Celleste software suite (Thermo Scientific).

### Integration site analysis

CypA^−/−^ (44), RANBP2_∆Cyp_, or control HT1080 cells stably transduced with doxycycline-inducible MX2 were infected with DNase-treated (Turbo DNase, Thermo Fisher) U3-tagged HIV-1 at a multiplicity of infection of 1. Three days post-infection, genomic DNA was extracted using the Machery-Nagel NucleoSpin Tissue Kit. Integration libraries were prepared using ligation-mediated PCR (LM-PCR) essentially as previously described (33, 62, 63). Genomic DNA (5 µg) was digested overnight with a cocktail of enzymes (100 U each, *AvrII*, *NheI-HF*, *SpeI-HF*, and *BamHI-HF*) and purified using a PCR purification kit (GeneJET). Following overnight ligation (four parallel reactions) to asymmetric linkers, DNA was purified again using a PCR purification kit. The ligated samples were subjected to two rounds of LM-PCR using virus and linker-specific primers (Data Set S1). Following PCR purification, libraries were assessed for fragment size distribution by TapeStation-4150, quantified by DNA fluorimetry (Qubit), and then pooled at 10 nM. Pooled samples were further diluted to 2 nM in Illumina sequencing resuspension buffer (RSB; Ref#20762979). Next, PhiX Control v3 DNA was spiked at 30%–40%, and the sample was diluted to 650 pM with RSB. The mixture (20 µL) was loaded into a P2 300 cycle cartridge and sequenced on an Illumina NextSeq 2000 sequencer.

Raw fastq files were demultiplexed using the Sabre tool (90) or by a Perl script. Post demultiplexing, files were trimmed, aligned to human genome build hg19, and bed files were generated as described (61). Integration into genes and SPADs was scored as within these genomic coordinates. For TSSs, CpG islands, and LADs, sites were mapped within ±2.5 kb windows (5 kb surrounding these coordinates). Gene density was assessed as number of genes per Mb. Random integration controls were generated by shearing hg19 *in silico* using the restriction enzyme sites used to generate the wet-bench samples and then mapping the resultant fragments with respect to the aforementioned genomic annotations (63).

### Statistical analyses

Statistical significance for CA binding and infectivity assays was determined using GraphPad software (two-way analysis of variance or *t* tests where appropriate). Comparison of percent integration was analyzed by Fisher’s exact test; comparison of gene density was analyzed by Wilcoxon rank-sum test. Statistical analyses not included in figures are included in Data Set S2.

## Data Availability

Illumina FASTQ raw reads are available at the National Center for Biotechnology Sequences Read Archive (accession number: BioProject PRJNA1147214).

## SUPPLEMENTAL MATERIAL

Data Set S1 mbio.02646-24-s0001.xlsx Raw data and oligonucleotide sequences for Fig. 4.

Data Set S2 mbio.02646-24-s0002.xlsx Statistical analyses not included in figures (Fig. 3B, S4, and S6).

Supplemental Material mbio.02646-24-s0003.pdf Figures S1 to S6; Tables S1 to S3.

ASM does not own the copyrights to Supplemental Material that may be linked to, or accessed through, an article. The authors have granted ASM a non-exclusive, world-wide license to publish the Supplemental Material files. Please contact the corresponding author directly for reuse.
