# Supplementary material for: Interplay between the cyclophilin homology domain of RANBP2 and MX2 regulates HIV-1 capsid dependencies on nucleoporins
Source: mBio. 2025 Jan 24;16(3):e02646-24. doi: 10.1128/mbio.02646-24 (PMC11898759; doi:10.1128/mbio.02646-24)
Supplement: Supplemental Material — Figures S1 to S6; Tables S1 to S3. [file mbio.02646-24-s0003.pdf]

Supplemental Materials

Interplay between the cyclophilin homology domain of RANBP2 and MX2 regulates HIV-1 capsid dependencies on nucleoporins

Haley Flick[1,6], Ananya Venbakkam[1,4,6], Parmit K. Singh[2,3], Bailey Layish[1], Szu-Wei Huang[5], Rajalingam Radhakrishnan[2], Mamuka Kvaratskhelia[5], Alan N. Engelman[2,3,4], Melissa Kane[1,4*]

Melissa Kane
Email: kaneme@pitt.edu

This PDF file includes:

Figures S1 to S6
Tables S1 to S3

Other supporting materials for this manuscript include the following:

Dataset S1
Dataset S2

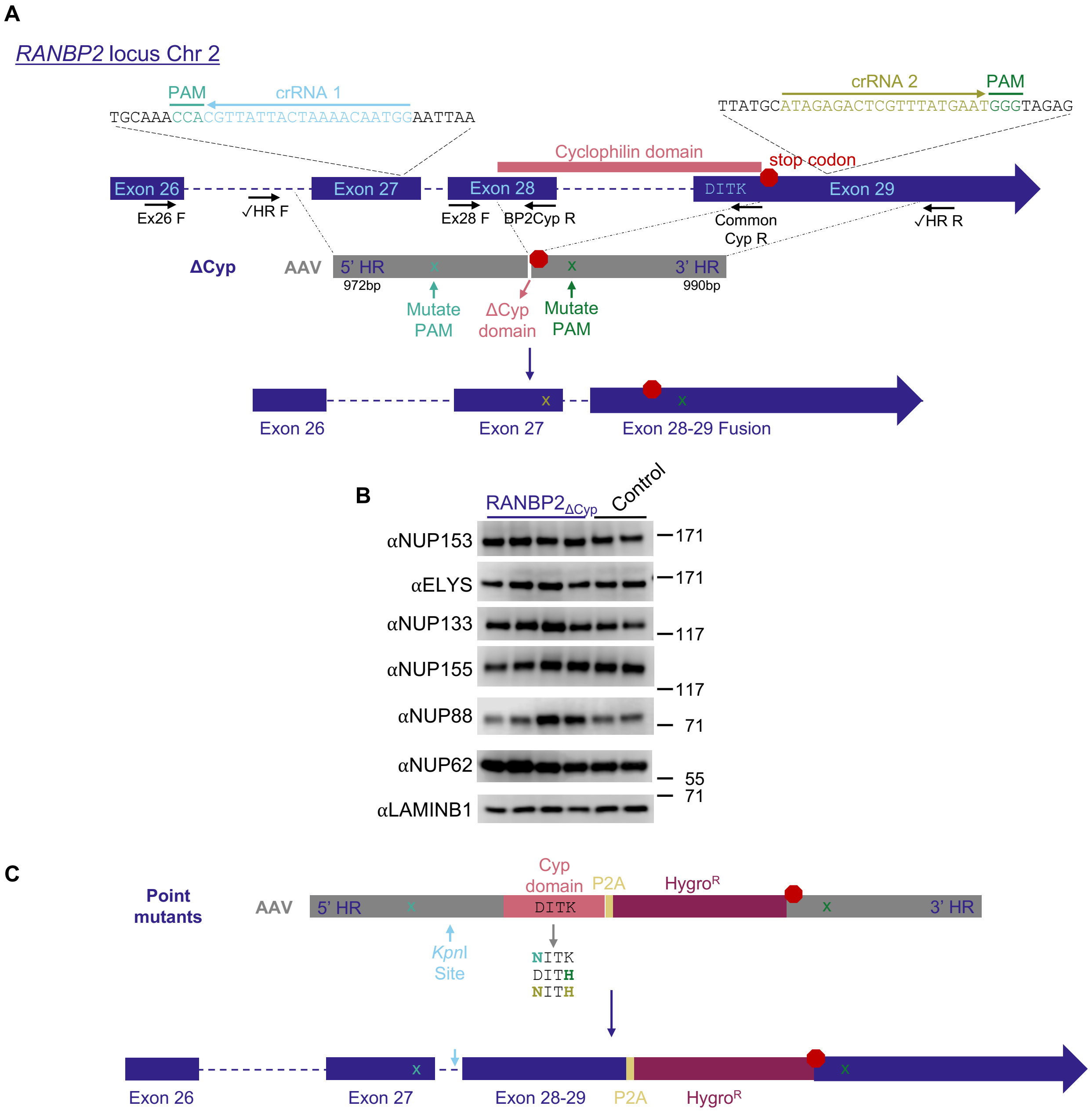

Fig. S1. Generation of RANBP2 mutant cell lines

A) Top - Schematic of the 3’ end of the *RANBP2* locus on Chromosome 2 including location of the cyclophilin (Cyp) domain, residues 3126-3129, and stop codon indicated; crRNA guide targeting sites exon 27 and exon 29, and PCR primers for mutant clone screening and verification indicated. Diagram of AAV donor for homology-directed repair for generation of RANBP2$_{\Delta Cyp}$ cells shown below. Bottom – Schematic of the 3’ of the locus following homology-directed repair with exons 28-29 fused and Cyp domain removed. B) Western blot analysis of Nup expression in control and mutant cell clones and LAMINB1 loading control. C) Top - Diagram of AAV donor for generation of RANBP2 point mutant and control cells with silent *KpnI* site and amino acid residues in wild-type and mutant RANBP2 cells indicated. Bottom - Schematic of the 3’ of the locus following homology-directed repair with exons 28-29 fused followed by P2A skipping site and Hygromycin-resistance gene (Hygro^R), the *KpnI* site in intron 27 indicated with a blue arrow.

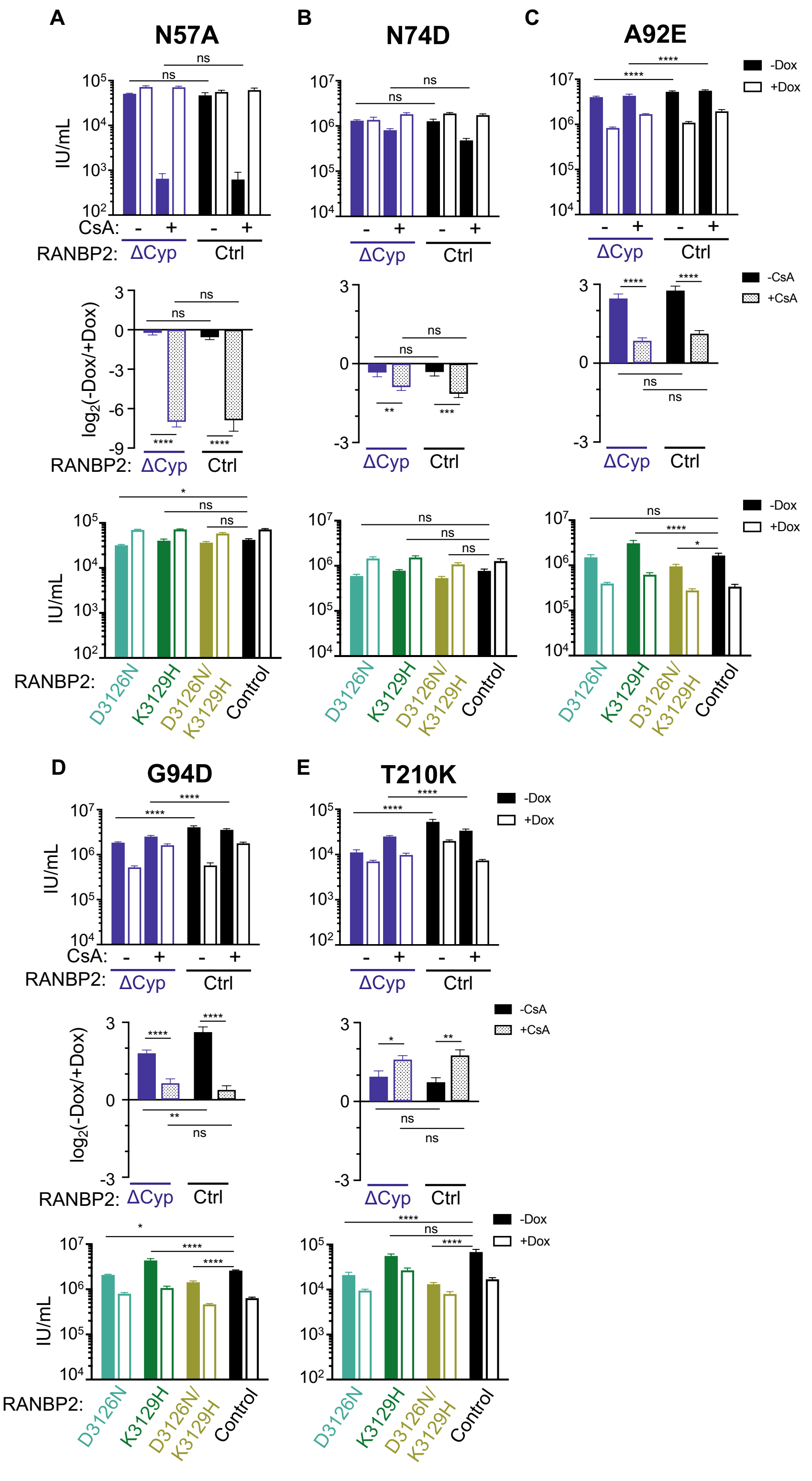

Fig. S2. Effects of the RanBP2-Cyp domain on HIV-1 CA mutant viral infection and Mx2 sensitivity.

A-E) (Top) Infection of control and $RANBP2_{\Delta Cyp}$ HT1080 cell clones stably transduced with doxycycline-inducible MX2 in the presence (open bars) or absence (filled bars) of doxycycline and presence or absence of CsA with the indicated GFP reporter viruses. Titers are represented as mean + sem of infectious units

(IU) per mL. $RANBP2_{\Delta Cyp}$: n≥12 technical replicates combined from ≥3 different clones; control: n≥8 technical replicates combined from ≥2 different clones; representative of ≥4 independent experiments. Statistical significance was determined by two-way ANOVA (Śídák's multiple comparisons test).
(Middle) Data from (top) shown as a ratio [fold change of -Dox(-MX2)/+Dox(+MX2)] in the presence (dotted bars) or absence (filled bars) of CsA. Average fold change calculated from three-four technical replicates per experiment; shown is mean + sem of $\log_2$(fold change) from four-eight independent experiments. Statistical significance was determined by paired *t* test.
(Bottom) Infection of control and RANBP2 point mutant HT1080 cell clones stably transduced with doxycycline-inducible MX2 in the presence (open bars) or absence (filled bars) of doxycycline with the indicated GFP reporter viruses. Titers are represented as mean + sem of infectious units (IU) per mL. n≥12 technical replicates combined from ≥4 independent experiments. Statistical significance was determined by two-way ANOVA (Śídák's multiple comparisons test)
ns, not significant (P≥0.05); * $P < 0.05$; ** $P < 0.01$; *** $P < 0.001$; **** $P < 0.0001$.

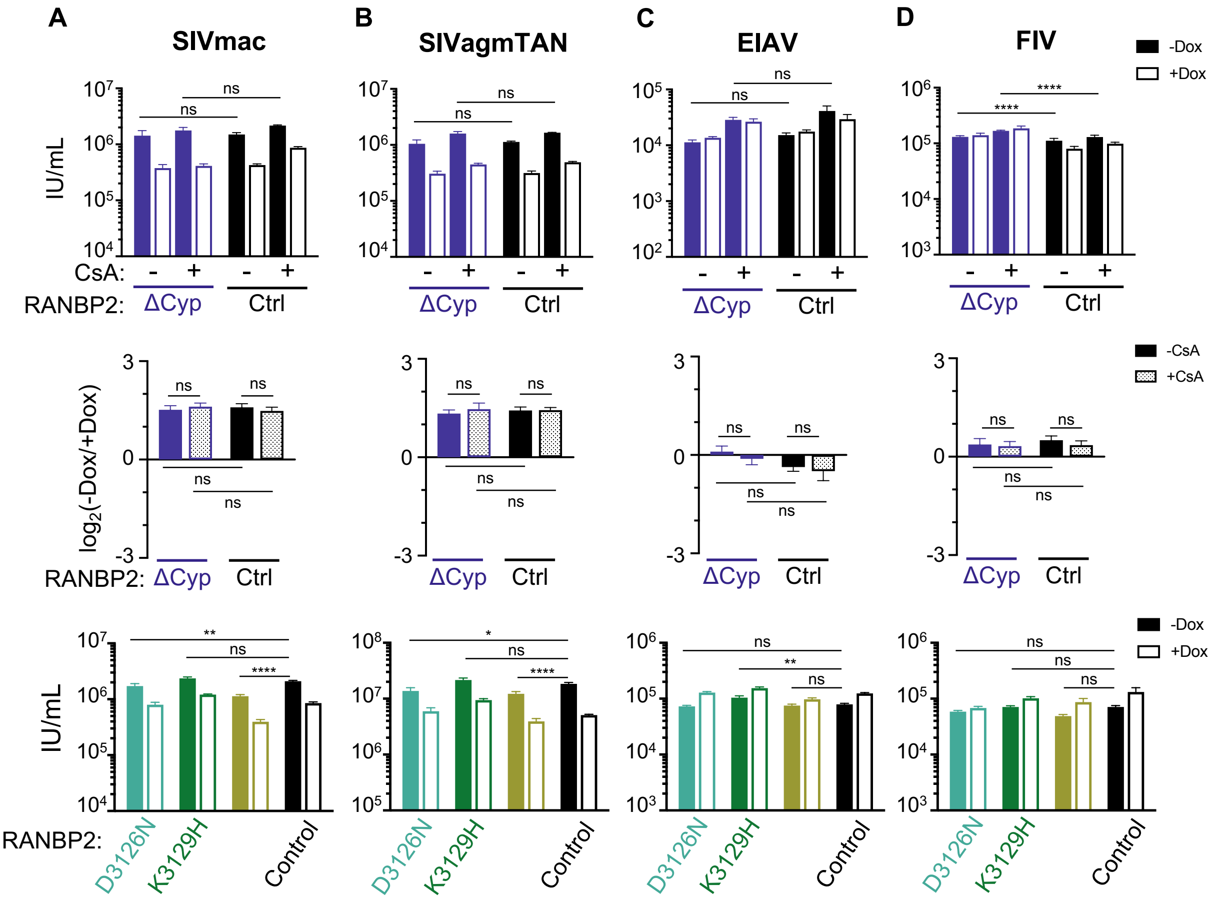

Fig. S3. Effects of the RanBP2-Cyp domain on lentivirus infection and Mx2 sensitivity.

A-D) (Top) Infection of control and RANBP2$_{\Delta Cyp}$ HT1080 cell clones stably transduced with doxycycline-inducible MX2 the presence (open bars) or absence (filled bars) of doxycycline and presence or absence of CsA with the indicated GFP reporter viruses. Titers are represented as mean + sem of infectious units (IU) per mL. RANBP2$_{\Delta Cyp}$: n≥12 technical replicates combined from ≥3 different clones; control: n≥8 technical replicates combined from ≥2 different clones; representative of ≥4 independent experiments. Statistical significance was determined by two-way ANOVA (Śídák's multiple comparisons test).

(Middle) Data from (top) shown as a ratio [fold change of -Dox(-MX2)/+Dox(+MX2)] in the presence (dotted bars) or absence (filled bars) of CsA. Average fold change calculated from three-to-four technical replicates per experiment; shown is mean + sem of log$_2$(fold change) from four-eight independent experiments. Statistical significance was determined by paired *t* test.

(Bottom) Infection of control and RANBP2 point mutant HT1080 cell clones stably transduced with doxycycline-inducible MX2 the presence (open bars) or absence (filled bars) of doxycycline with the indicated GFP reporter viruses. Titers are represented as mean + sem of infectious units (IU) per mL. n≥12 technical replicates combined from ≥4 independent experiments. Statistical significance was determined by two-way ANOVA (Śídák's multiple comparisons test).

ns, not significant ($P \geq 0.05$); * $P < 0.05$; ** $P < 0.01$; *** $P < 0.001$; **** $P < 0.0001$.

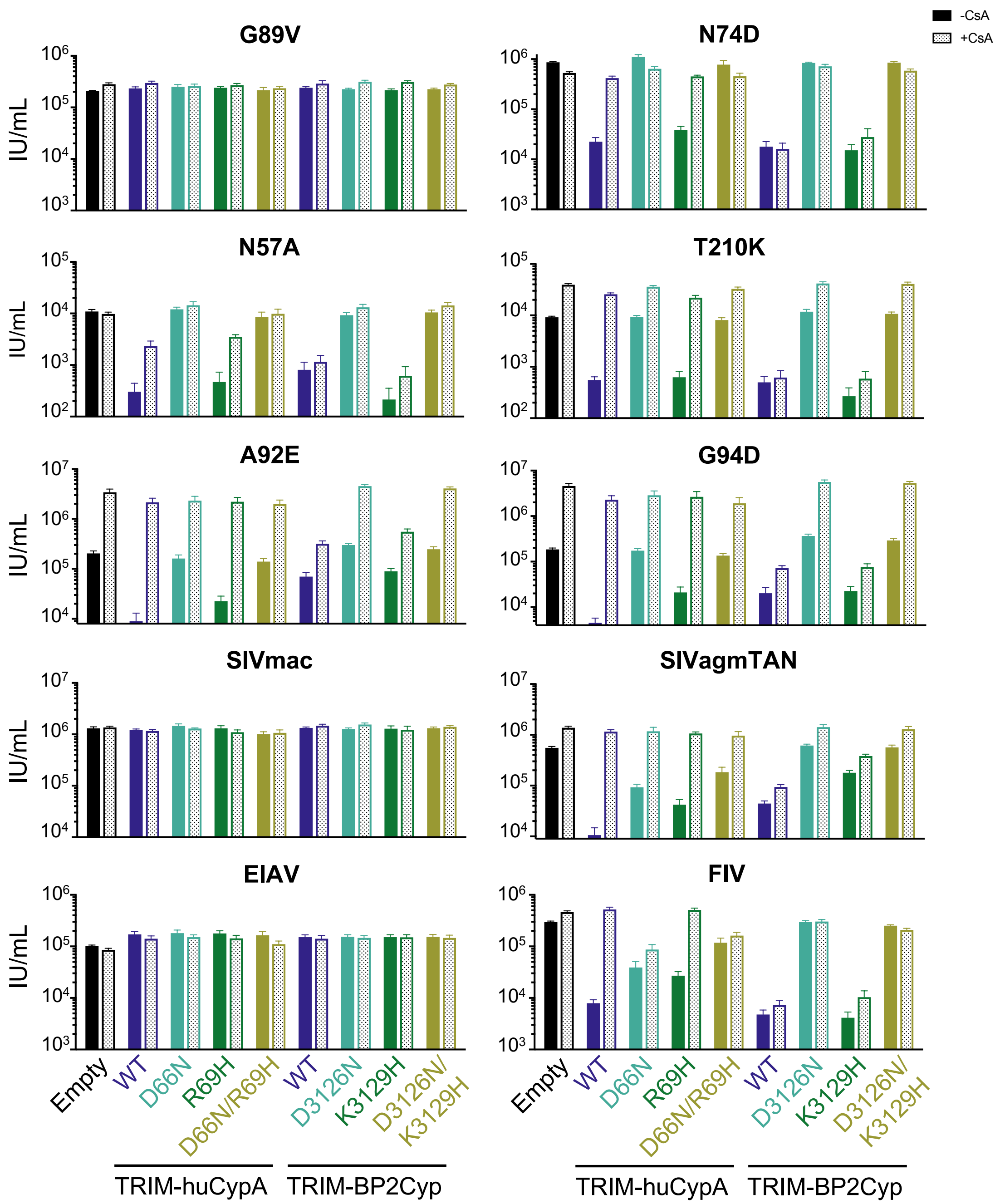

Fig. S4. Recognition of lentiviruses and HIV-1 CA mutants by TRIM-Cyp fusions.
Infectivity of GFP reporter viruses on HeLa cells stably expressing control empty vector, or chimeras of the TRIM5 N-terminal domain of owl monkey TRIMCyp with human cyclophilin A (huCypA), human $CypA_{D66N}$, $CypA_{R69H}$, $CypA_{D66N/R69H}$ mutants, human RANBP2-Cyp (BP2-Cyp), or $BP2\text{-}Cyp_{D3126N}$, $BP2\text{-}Cyp_{K3129H}$, $BP2\text{-}Cyp_{D3126N/K3129H}$ mutants. Titers are represented as mean + sem of infectious units (IU) per mL, n≥12 technical replicates combined from ≥3 independent experiments. Statistical analysis in Dataset S2.

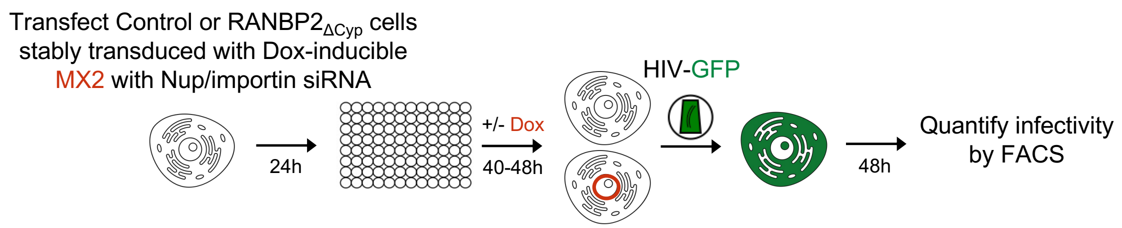

Fig. S5. Experimental design for Nup/NTR knockdown
Experimental strategy to investigate the roles of Nups and NTRs in HIV infection and the antiviral activity of MX2. For a detailed description, refer to the Methods.

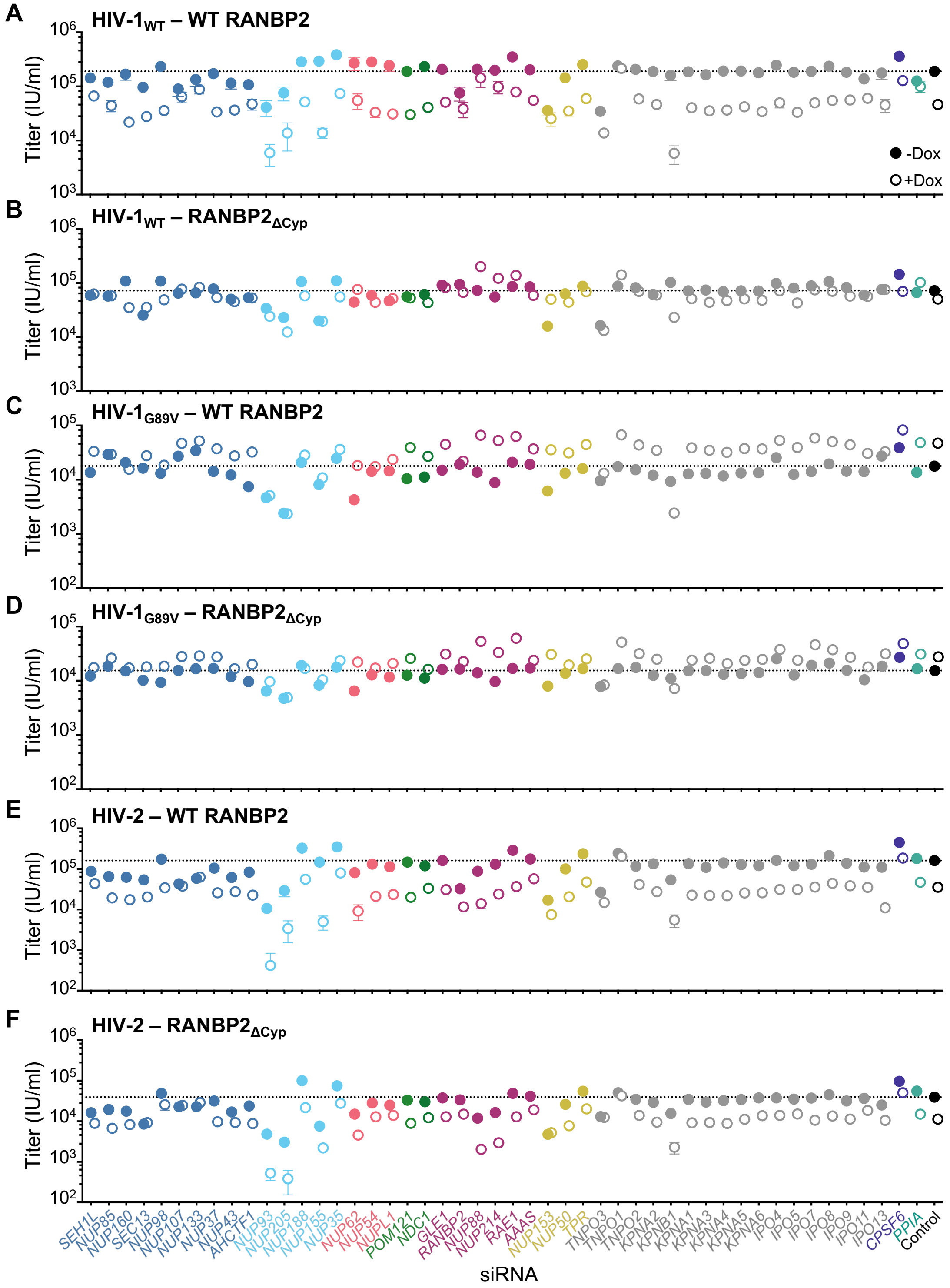

Fig. S6. Effect of RANBP2-Cyp on the nucleoporin requirements for HIV infection and MX2 sensitivity.

Infectivity of HIV GFP reporter virus in control (A,C,E) and RANBP2$_{\Delta Cyp}$ (B,D,F) HT1080 cells stably transduced with doxycycline-inducible MX2 in the presence (open circles) or absence (filled circles) of doxycycline 64-72 h after transfection with siRNA (color coded by subcomplex as in Fig. 5A). Titers are mean ± sem, n=8 technical replicates from one control and one RANBP2$_{\Delta Cyp}$ clone from two independent experiments. Statistical analysis in Dataset S2.

Table S1. Oligonucleotides for Plasmid Construction

**TRIM5-fusions**	
BP2-Cyp NotI F	ataagaatgcggccgccAATCCTGTGGTGTTTTTTGATG
BP2-Cyp SalI R	ataagaatgtcgactcaTATCTGTCCACATTCTGTG
BP2-Cyp-DtoN-F	GTTTGCCAAGGAGGAAATATCACCAAACATGATGGAACAGG
BP2-Cyp-DtoN-R	CCTGTTCCATCATGTTTGGTGATATTTCCTCCTTGGCAAAC
BP2-Cyp-KtoH-F	GTTTGCCAAGGAGGAGATATCACCCATCATGATGGAACAGG
BP2-Cyp-KtoH-R	CCTGTTCCATCATGATGGGTGATATCTCCTCCTTGGCAAAC
BP2-Cyp-NH-F	GTTTGCCAAGGAGGAAATATCACCCATCATGATGGAACAGG
BP2-Cyp-NH-R	CCTGTTCCATCATGATGGGTGATATTTCCTCCTTGGCAAAC
**AAV**	
AAV NheI F	TTCCTGCGGCCGCAGCATAGCTAGC
AAV XhoI R	CGCTCGGTCCGCACAATTCCTCGAG
BP2-Cyp AAV KpnI F	AATACTAAGGTACCTGCTTCCCC
BP2-Cyp AAV NdeI R	TGAACAACATATGATTTTATATCTGTCCACATTC
**RANBP2-mutant cell screening and verification**	
BP2delCyp Check HR F	TGGAAGCTCTATTTATTAGATGTCTAGC
BP2delCyp Check HR R	CTTTGTAGTTCCATATTGAGTAGGCC
BP2 Exon 26 F1	GAACCCAGTCAGCCGGTAAA
BP2Cyp R	AACACCACAGGATTGGTCTCC
BP2 Exon 28 F	ATGGAGAAGCAAAAGTAGAAC
Common Cyp R2	TTTTCATCTTCAAATTTGTC

Table S2. ON-TARGET SMARTpool siRNA utilized in this investigation

Gene Symbol	Gene ID
AAAS	8086
AHCTF1	25909
CPSF6	11052
GLE1	2733
IPO11	51194
IPO13	9670
IPO4	79711
IPO5	3843
IPO7	10527
IPO8	10526
IPO9	55705
KPNA1	3836
KPNA2	3838
KPNA3	3839
KPNA4	3840
KPNA5	3841
KPNA6	23633
KPNB1	3837
NDC1	55706
NUP107	57122
NUP133	55746
NUP153	9972
NUP155	9631
NUP160	23279
NUP188	23511
NUP205	23165
NUP210	23225
NUP214	8021
NUP35	129401
NUP37	79023
NUP43	348995
NUP50	10762
NUP54	53371
NUP62	23636
NUP85	79902
NUP88	4927
NUP93	9688
NUP98	4928
NUPL1	9818
POM121	9883
PPIA	5478
RAE1	8480
RANBP2	5903
SEC13	6396
SEH1L	81929
TNPO1	3842
TNPO2	30000
TNPO3	23534
TPR	7175

Table S3. Antibodies utilized in this investigation

Reactivity	Species	Company	Catalog Number
GAPDH	mouse	Santa Cruz	sc-47724
HIV-1 p55+p24+p17	rabbit	Abcam	ab63917
MX2	rabbit	Novus Biologicals	NBP1-8108
RANBP2	rabbit	Abcam	Ab64276
NUP153	mouse	Proteintech	Ab96462
ELYS	mouse	Abcam	Ab53540
NUP133	mouse	Santa Cruz	376763
NUP155	rabbit	Abcam	Ab157104
NUP88	mouse	BD Biosciences	611896
NUP62	mouse	BD Biosciences	610498
HA tag	mouse	BioLegend	901514
LAMIN B1	Rabbit	Abcam	Ab133741
Tubulin	mouse	Sigma-Aldrich	T6074

Dataset S1 (separate file). Integration Site Analysis
Raw data and oligonucleotide sequences for Fig. 4 are included in this file.

Dataset S2 (separate file). Statistical Analysis
Statistical analyses not included in figures (Figs. 3B, S4, and 5/S6) is detailed in this file.
